# Preoperative Chemoradiotherapy vs Chemotherapy for Adenocarcinoma of the Esophagogastric Junction: A Network Meta-Analysis

**DOI:** 10.1001/jamanetworkopen.2024.25581

**Published:** 2024-08-02

**Authors:** Ulrich Ronellenfitsch, Juliane Friedrichs, Emilie Barbier, Gary A. Bass, Bryan Burmeister, David Cunningham, Ben M. Eyck, Maurizio Grilli, Ralf-Dieter Hofheinz, Meinhard Kieser, Jörg Kleeff, Fredrik Klevebro, Ruth Langley, Florian Lordick, Manfred Lutz, Murielle Mauer, Christoph W. Michalski, Patrick Michl, Matthew Nankivell, Magnus Nilsson, Svenja Seide, Manish A. Shah, Qian Shi, Michael Stahl, Susan Urba, Jan van Lanschot, Dirk Vordermark, Thomas Noel Walsh, Marc Ychou, Tanja Proctor, Johannes A. Vey

**Affiliations:** 1 Department of Abdominal, Vascular and Endocrine Surgery, Medical Faculty of the Martin-Luther-University Halle-Wittenberg and University Hospital Halle (Saale), Halle (Saale), Germany; 2 Fédération Francophone de Cancérologie Digestive, Centre de Recherche Institut, Institut National de la Santé et de la Recherche Médicale, Epidemiology of Digestive Cancers, University of Burgundy, Franche-Comté, France; 3 Division of Traumatology, Surgical Critical Care and Emergency Surgery, University of Pennsylvania, Philadelphia; 4 Department of Radiation Oncology, GenesisCare Fraser Coast and the Hervey Bay Hospital, Urraween, Australia; 5 Institute of Cancer Research, National Institute for Health and Care Research Biomedical Research Centre, The Royal Marsden Hospital, London, United Kingdom; 6 Department of Surgery, Erasmus MC Cancer Institute, Rotterdam, the Netherlands; 7 Library of the Medical Faculty Mannheim, Heidelberg University, Mannheim, Germany; 8 Day Treatment Center, Interdisciplinary Tumor Center Mannheim and Third Department of Internal Medicine, University Medical Centre Mannheim, University of Heidelberg, Mannheim, Germany; 9 Institute of Medical Biometry, University of Heidelberg, Heidelberg, Germany; 10 Department of Clinical Science, Intervention and Technology, Karolinska Institute, Center for Upper Gastrointestinal Diseases, Karolinska University Hospital, Stockholm, Sweden; 11 MRC (Medical Research Council) Clinical Trials Unit, University College London, London, United Kingdom; 12 Department of Oncology, University Cancer Center Leipzig and Cancer Center Central Germany, University of Leipzig Medical Center, Leipzig, Germany; 13 Department of Gastroenterology, Endocrinology, and Infectiology, Caritasklinik St Theresia, Saarbrücken, Germany; 14 Statistics Department, European Organisation for Research and Treatment of Cancer, Brussels, Belgium; 15 Department of General, Abdominal and Transplant Surgery, University Hospital Heidelberg, Heidelberg, Germany; 16 Department of Gastroenterology, Infectiology and Toxicology, University Hospital Heidelberg, Heidelberg, Germany; 17 Boehringer Ingelheim, Ingelheim, Germany; 18 Solid Tumor Oncology, Weill Cornell Medicine, New York, New York; 19 Division of Clinical Trials and Biostatistics, Department of Quantitative Health Sciences, Mayo Clinic, Rochester, Minnesota; 20 Department of Medical Oncology and Hematology With Integrated Palliative Medicine, Protestant Hospital Essen-Mitte, Essen, Germany; 21 Department of Internal Medicine, Division of Hematology/Oncology, University of Michigan, Ann Arbor; 22 Department of Radiotherapy, Medical Faculty of the Martin-Luther-University Halle-Wittenberg and University Hospital Halle (Saale), Halle (Saale), Germany; 23 Royal College of Surgeons in Ireland, Dublin; 24 Montpellier Cancer Institute, Montpellier, France

## Abstract

**Importance:**

The prognosis of patients with adenocarcinoma of the esophagus and esophagogastric junction (AEG) is poor. From current evidence, it remains unclear to what extent preoperative chemoradiotherapy (CRT) or preoperative and/or perioperative chemotherapy achieve better outcomes than surgery alone.

**Objective:**

To assess the association of preoperative CRT and preoperative and/or perioperative chemotherapy in patients with AEG with overall survival and other outcomes.

**Data Sources:**

Literature search in PubMed, Cochrane Library, Cumulative Index to Nursing and Allied Health Literature, ClinicalTrials.gov, and International Clinical Trials Registry Platform was performed from inception to April 21, 2023.

**Study Selection:**

Two blinded reviewers screened for randomized clinical trials comparing preoperative CRT plus surgery with preoperative and/or perioperative chemotherapy plus surgery, 1 intervention with surgery alone, or all 3 treatments. Only data from participants with AEG were included from trials that encompassed mixed histology or gastric cancer. Among 2768 initially identified studies, 17 (0.6%) met the selection criteria.

**Data Extraction and Synthesis:**

The Preferred Reporting Items for Systematic Reviews and Meta-analyses (PRISMA) reporting guidelines were followed for extracting data and assessing data quality by 2 independent extractors. A bayesian network meta-analysis was conducted using the 2-stage approach.

**Main Outcomes and Measures:**

Overall and disease-free survival, postoperative morbidity, and mortality.

**Results:**

The analyses included 2549 patients (2206 [86.5%] male; mean [SD] age, 61.0 [9.4] years) from 17 trials (conducted from 1989-2016). Both preoperative CRT plus surgery (hazard ratio [HR], 0.75 [95% credible interval (CrI), 0.62-0.90]; 3-year difference, 105 deaths per 1000 patients) and preoperative and/or perioperative chemotherapy plus surgery (HR, 0.78 [95% CrI, 0.64-0.91]; 3-year difference, 90 deaths per 1000 patients) showed longer overall survival than surgery alone. Comparing the 2 modalities yielded similar overall survival (HR, 1.04 [95% CrI], 0.83-1.28]; 3-year difference, 15 deaths per 1000 patients fewer for CRT). Similarly, disease-free survival was longer for both modalities compared with surgery alone. Postoperative morbidity was more frequent after CRT plus surgery (odds ratio [OR], 2.94 [95% CrI, 1.01-8.59]) than surgery alone. Postoperative mortality was not significantly more frequent after CRT plus surgery than surgery alone (OR, 2.50 [95% CrI, 0.66-10.56]) or after chemotherapy plus surgery than CRT plus surgery (OR, 0.44 [95% CrI, 0.08-2.00]).

**Conclusions and Relevance:**

In this meta-analysis of patients with AEG, both preoperative CRT and preoperative and/or perioperative chemotherapy were associated with longer survival without relevant differences between the 2 modalities. Thus, either of the 2 treatments may be recommended to patients.

## Key Points

**Question:**

Is preoperative chemoradiotherapy (CRT) or preoperative and/or perioperative chemotherapy associated with improved outcomes in patients with adenocarcinoma of the esophagus and esophagogastric junction (AEG)?

**Findings:**

In this network meta-analysis consisting of individual data from 2549 patients in 17 studies, both preoperative CRT plus surgery and preoperative and/or perioperative chemotherapy plus surgery were associated with longer overall survival compared with surgery alone in patients with AEG.

**Meaning:**

These results suggest that preoperative CRT and preoperative and/or perioperative chemotherapy are both associated with longer survival for patients with AEG.

## Teaser

This individual patient data network meta-analysis assesses the association between preoperative chemoradiotherapy and preoperative and/or perioperative chemotherapy compared with surgery alone in patients with adenocarcinoma of the esophagus and esophagogastric junction.

## Introduction

In 2020, esophageal cancer ranked sixth in mortality (544 100 deaths) worldwide.^1,2^ It consists of squamous cell carcinoma and adenocarcinoma. Esophageal and esophagogastric junction adenocarcinoma are considered a single entity, adenocarcinoma of the esophagogastric junction (AEG). The prognosis for patients with AEG undergoing upfront surgery has been poor. Five-year survival ranged from 36.9% for patients with node-negative disease to 9.6% for those with node-positive disease.^2^

Substantial evidence suggests that preoperative chemotherapy or chemoradiotherapy (CRT) prolongs overall survival (OS) compared with surgery alone.^3,4,5^ While preoperative CRT is usually not continued postoperatively, chemotherapy is given preoperatively and postoperatively (perioperative chemotherapy). Preoperative is preferred to mere postoperative treatment because it increases the likelihood of complete resection. In addition, many patients are unable to begin or sustain postoperative treatment due to complications or deterioration.^6,7^

The available evidence does not allow a conclusion on whether preoperative CRT or preoperative and/or perioperative chemotherapy has better outcomes for AEG. Both have shown prolonged survival compared with surgery alone in randomized clinical trials (RCTs)^4,8,9,10,11,12^ and meta-analyses.^3,5,13^ A randomized head-to-head comparison has been performed in 4 trials, with 3 showing inconclusive results,^14,15,16^ and the large Neo-AEGIS trial (Neoadjuvant Trial in Adenocarcinoma of the Oesophagus and Oesophagogastric Junction International Study)^17^ reporting similar survival and quality of life between treatment groups, thus suggesting equipoise. Three other RCTs directly comparing the modalities^18,19,20^ have not reported results yet. In summary, it remains unclear which is the best multimodal approach for treating AEG. To integrate the evidence comparing preoperative CRT, preoperative and/or perioperative chemotherapy, and surgery alone with regard to relevant outcomes in patients with AEG, we performed an individual patient data (IPD) network meta-analysis (NMA) including data from all pertinent RCTs.

## Methods

The work was conducted according to Preferred Reporting Items for Systematic Reviews and Meta-analyses (PRISMA) reporting guidelines. It was registered and the protocol published in the Cochrane Library.^21^ It was approved by the ethics committee of the Medical Faculty, Martin-Luther-University Halle-Wittenberg, Halle, Germany, with a waiver of informed consent because data were provided anonymously.

### Inclusion Criteria and Literature Search

We included patients from RCTs comparing at least 2 of the following: preoperative CRT plus surgery, preoperative and/or perioperative chemotherapy plus surgery, or surgery alone. Participants needed to have nonmetastatic, untreated, resectable AEG. There were no restrictions regarding blinding, follow-up, study size, and language. We searched the following databases from inception to April 21, 2023, using a predefined search strategy (eAppendix 1 in Supplement 1): PubMed, Cochrane Library, Cumulative Index to Nursing and Allied Health Literature, ClinicalTrials.gov, and International Clinical Trials Registry Platform. We checked reference lists of included studies for additional references.

### Literature Screening and Data Collection

Two reviewers (U.R. and J.F.) independently screened titles, abstracts, and, if potentially eligible, full texts for inclusion. Disagreement was resolved by a third reviewer (J.K.). Individual patient data were requested from all trials for all randomized participants fulfilling inclusion criteria. For trials not providing IPD, aggregate data (AD) were extracted by 2 researchers independently (U.R. and J.F.).

### Data Quality and Risk of Bias

Data quality checks were performed (eAppendix 2 in Supplement 1). Two researchers (U.R. and J.F.) independently assessed risk of bias for each included study using criteria outlined in the Cochrane Handbook for Systematic Reviews of Interventions^22^ and version 2 of the Cochrane Risk of Bias 2 tool^23^ (eAppendix 3 in Supplement 1).

### Variables

Individual patient data were requested or AD were retrieved for patient and trial characteristics (eAppendix 4 in Supplement 1). Outcomes included OS (randomization until death), disease-free survival (DFS; from a landmark 6 months after randomization until recurrence or death), local recurrence-free survival (RFS; from a landmark 6 months after randomization until local recurrence), distant RFS (from a landmark 6 months after randomization until distant recurrence), toxicity, postoperative mortality or morbidity, microscopically tumor-free (R0) resection margin, pT category at resection, pathological complete response (pCR), and quality of life. Information on race and ethnicity was not available from IPD or AD.

### Statistical Analysis

Network graphs were created with nodes representing interventions, edges representing treatment comparisons, and line thickness proportional to the number of trials comparing 2 treatments. The NMAs were conducted using the 2-stage approach.^24,25^ In the first stage, relative treatment effects were estimated from IPD, or AD if IPD were unavailable, for each study separately. For survival, the log–hazard ratio (HR) with SE was calculated per study applying a Cox proportional hazards regression model with the log-HR adjusted for age and sex. Prior death, recurrence, or failure to become disease free were regarded as events at the landmark used for DFS and local and distant RFS analyses. A logistic regression model was applied for estimating the log–odds ratio (OR) with SE of the binary outcomes for each study. Unadjusted ORs were estimated, because reported ORs of the studies not providing IPD were not adjusted. For the analyses of postoperative mortality, morbidity, and tumor stage, only patients who underwent surgery were included.

In the second stage, the estimated treatment effects were combined by applying a bayesian random-effects model using weakly informative half-normal priors for heterogeneity and a vague prior for treatment effects (eAppendix 5 in Supplement 1).^26,27,28^ Computations were done on the log scale, and results were transformed back for presenting pooled HRs and ORs with 95% credible intervals (CrIs). For OS and DFS, anticipated absolute effects were computed as absolute risk for an event occurring within 3 years using the estimated HRs.

For each comparison, consistency of the evidence was assessed by the node-splitting approach.^29,30^ The available evidence in the network was split at a node and the direct and indirect estimates of the treatment effect were assessed for agreement (eAppendix 5 in Supplement 1). Heterogeneity was measured as a τ value representing the SD of the underlying effects across studies. Treatment ranking was performed by calculating the surface under the cumulative ranking (SUCRA) curve from the posterior probability of being the most successful treatment.^31^ A SUCRA value of 1.00 indicates a treatment certain to be the best; a value of 0, certain to be the worst.^31^ Additionally, the median rank and 95% CrI of the posterior distribution for the rank were calculated.

Subgroup analyses for OS and DFS were conducted by using the bayesian NMA approach described earlier (eAppendix 6 in Supplement 1). Sensitivity analyses were conducted for all outcomes with respect to model assumptions and the choice of priors to investigate robustness of network results.^32^ All analyses were performed using R, version 4.4.0 (R Project for Statistical Computing)^33^ and JAGS, version 4.3.1 (SourceForce).^34^ For inconsistency tests, 2-sided *P* < .05 indicated statistical significance.

## Results

The analyses included 2549 patients (mean [SD] age, 61.0 [9.4] years; 2206 (86.5%) male and 343 [13.5%] female). A total of 1255 patients (68.5% of those with information available) had an Eastern Cooperative Oncology Group performance status of 0, and 1314 (65.1% of those with information available) had AEG type I.

### Study Selection and Study Details

**Figure 1.**
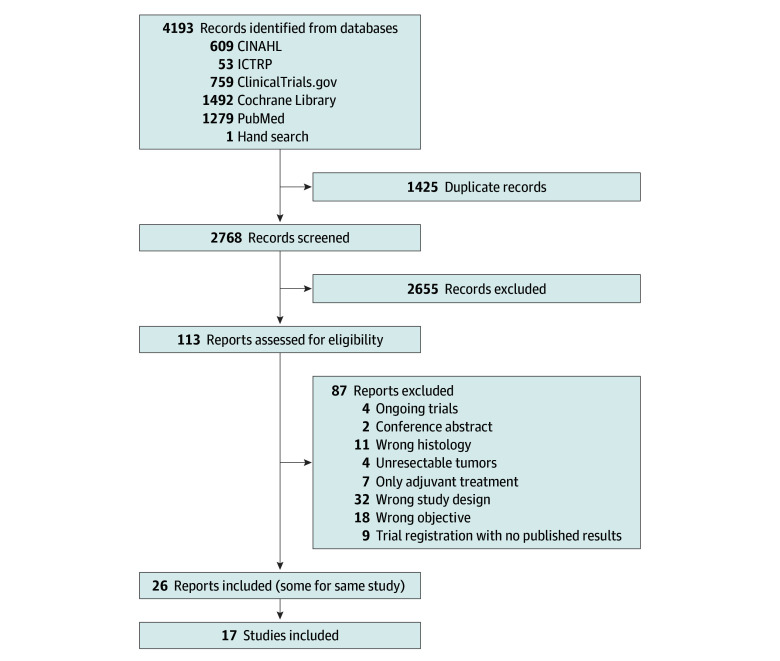
PRISMA Flow Diagram CINAHL indicates Cumulative Index to Nursing and Allied Health Literature; ICTRP, International Clinical Trials Registry Platform.

The literature search yielded 4193 records (Figure 1). After excluding duplicates, 2768 records were screened. Seventeen trials^4,8,9,10,11,12,14,15,16,19,35,36,37,38,39,40,41^ were included in the analyses (relevant excluded trials are listed in the eResults in Supplement 1). Eight trials^4,8,10,16,35,36,37,40^ included both squamous cell carcinoma and adenocarcinoma. From these, only participants with AEG were included. Five trials^8,9,12,36,38^ compared preoperative and/or perioperative chemotherapy plus surgery with surgery alone; 8 trials^4,10,11,35,37,39,40,41^ compared preoperative CRT plus surgery with surgery alone; and 4 trials^14,15,16,19^ compared preoperative and/or perioperative chemotherapy plus surgery with preoperative CRT plus surgery. In the trials with respective information available,^4,8,9^ 87.9% to 91.8% of randomized patients completed all planned preoperative CRT or chemotherapy cycles, whereas only 38.5% completed all planned perioperative chemotherapy cycles. Individual patient data were available from 14 trials^4,8,9,10,11,12,14,15,16,35,36,37,38,40^ and unavailable from 3 trials,^19,39,41^ of which 2 trials^19,41^ had not yet reported OS. Main trial characteristics are displayed in Table 1. Network graphs are shown in eFigure 1 in Supplement 1.

**Table 1.** Characteristics of the 17 Included Trials

Trial	Recruitment period and location	Included histology	Treatment scheme per group (No. of patients with AEG per trial group included in meta-analysis)
**IPD available**			
Ychou et al,^12^ 2011 (ACCORD)	1995-2003, France	Adenocarcinoma	Group A: cisplatin, 100 mg/m^2^, on day 1, fluorouracil, 800 mg/m^2^/d, on days 1-5, 2-3 preoperative and 3-4 postoperative cycles (n = 85); group B: surgery alone (n = 84)
Tepper et al,^10^ 2008 (CALGB 9781)	1997-2000, US	Adenocarcinoma and squamous cell carcinoma	Group A: preoperative simultaneous CRT, 50.4 Gy, cisplatin, 100 mg/m^2^, on days 1 and 29, fluorouracil, 1000 mg/m^2^, on days 1-4 and d 29-32 (n = 23); group B: surgery alone (n = 19)
Eyck et al,^4^2021 (CROSS)	2004-2008, the Netherlands	Adenocarcinoma and squamous cell carcinoma	Group A: preoperative simultaneous CRT, 41.4 Gy, and paclitaxel, 50 mg/m^2^, carboplatin (area under the curve, 2 mg/mL/min) (n = 134); group B: surgery alone (n = 141)
Schuhmacher et al,^38^ 2010 (EORTC 40954)	1999-2004, several European countries	Adenocarcinoma	Group A: cisplatin, 50 mg/m^2^, on days 1, 15, and 29, fluorouracil, 2000 mg/m^2^, on days 1, 8, 15, 22, and 36, 2 preoperative cycles (n = 37); group B: surgery alone (n = 39)
Mariette et al,^37^ 2014 (FFCD 9901)	2000-2009, France	Adenocarcinoma and squamous cell carcinoma	Group A: preoperative simultaneous CRT with 45 Gy, cisplatin, 75 mg/m^2^, on days 1 or 2 and 29 or 30, fluorouracil, 800 mg/m^2^, on days 1-4 and 29-32 (n = 30); group B: surgery alone (n = 27)
Cunningham et al,^9^ 2006 (MAGIC)	1994-2002, United Kingdom, the Netherlands, Germany, Singapore, New Zealand, and Brazil	Adenocarcinoma	Group A: epirubicin, 50 mg/m^2^, on day 1, cisplatin, 60 mg/m^2^, on day 1, fluorouracil, 200 mg/m^2^, on days 1 to 21, 3 preoperative and 3-4 postoperative cycles (n = 65); group B: surgery alone (n = 66)
von Döbeln et al,^16^ 2019 (NeoRes)	2006-2013, Sweden and Norway	Adenocarcinoma and squamous cell carcinoma	Group A: cisplatin, 100 mg/m^2^, day 1, fluorouracil, 750 mg/m^2^, days 1-5, 3 preoperative cycles (n = 66); group B: preoperative simultaneous CRT, 40 Gy, cisplatin, 100 mg/m^2^ on day 1, fluorouracil, 750 mg/m^2^, on days 1-5, 3 preoperative cycles (n = 65)
Allum et al,^8^ 2009 (OE02)	1992-1998, European countries	Adenocarcinoma and squamous cell carcinoma	Group A: cisplatin, 80 mg/m^2^, on day 1, fluorouracil, 1000 mg/m^2^, on days 1-4, 2 preoperative cycles (n = 265); group B: surgery alone (n = 268)
Stahl et al,^15^ 2017 (POET)	2000-2005, Germany	Adenocarcinoma	Group A: cisplatin, 50 mg/m^2^, biweekly, fluorouracil, 2000 mg/m^2^, weekly, 2.5 preoperative cycles (n = 59); group B: cisplatin, 50 mg/m^2^, biweekly, fluorouracil, 2000 mg/m^2^, weekly, 2 preoperative cycles followed by preoperative simultaneous CRT, 30 Gy, cisplatin, 50 mg/m^2^, on days 1-8, etoposide, 80 mg/m^2^, on days 3-5 (n = 60)
Kelsen et al,^36^ 2007 (RTOG 8911)	1990-1995, US and Canada	Adenocarcinoma and squamous cell carcinoma	Group A: cisplatin,100 mg/m^2^, on day 1, fluorouracil, 1000 mg/m^2^, on days 1-5, 2 preoperative cycles (n = 121); group B: surgery alone (n = 126)
Burmeister et al,^14^ 2011 (TROG)	2000-2006, Australia and New Zealand	Adenocarcinoma	Group A: cisplatin, 80 mg/m^2^, on day 1, fluorouracil, 1000 mg/m^2^, on days 1-4, 2 preoperative cycles (n = 38); group B: cisplatin, 80 mg/m^2^, on day 1, fluorouracil, 1000 mg/m^2^, on days 1-4, 2 preoperative cycles, simultaneous radiotherapy, 35 Gy, 15 fractions, with second cycle with fluorouracil reduced to 800 mg/m^2^ (n = 39)
Burmeister et al,^35^ 2005 (TROG AGITG)	2000-2006, Australia and New Zealand	Adenocarcinoma and squamous cell carcinoma	Group A: preoperative simultaneous CRT, 35 Gy, cisplatin, 80 mg/m^2^, on day 1, fluorouracil, 800 mg/m^2^, on days 1-4, 2 preoperative cycles (n = 80); group B: surgery alone (n = 78)
Urba et al,^40^ 2001	1989-1994, US	Adenocarcinoma and squamous cell carcinoma	Group A: preoperative simultaneous CRT, 45 Gy, cisplatin, 20 mg/m^2^, on days 1-5 and 17-21, fluorouracil, 300 mg/m^2^, on days 1-21, vinblastine, 1 mg/m^2^, on days 1-4 and 17-21 (n = 37); group B: surgery alone (n = 39)
Walsh et al,^11^ 1996	1990-1995, Ireland	Adenocarcinoma	Group A: preoperative simultaneous CRT, 40 Gy, cisplatin, 75 mg/m^2^, on days 7 and 49, fluorouracil, 15 mg/kg on days 1-5 and 42-47 (n = 58); group B: surgery alone (n = 55)
**IPD unavailable**			
Tian et al,^39^ 2021	2012-2016, China	Adenocarcinoma	Group A: preoperative simultaneous CRT, 45 Gy, capecitabine, 1000 mg/m^2^, twice daily on days 1-14, oxaliplatin, 130 mg/m^2^, on day 1, 2 preoperative cycles, 6 postoperative cycles (n = 76); group B: surgery alone (n = 73)
Leong et al,^19^ 2017	2009-2014, Australia, New Zealand, and Belgium, Germany, Canada	Adenocarcinoma	Group A: epirubicin, 50 mg/m^2^, on day 1, cisplatin, 60 mg/m^2^, on day 1, fluorouracil, 200 mg/m^2^ for 21-d continuous infusion, 3 preoperative cycles, 3 postoperative cycles (n = 60); group B: epirubicin, 50 mg/m^2^, on day 1, cisplatin, 60 mg/m^2^, on day 1, fluorouracil, 200 mg/m^2^, for 21-d continuous infusion, 2 preoperative cycles, simultaneous CRT, 45 Gy, continuous fluorouracil, 200 mg/m^2^, on days 1-25, epirubicin, 50 mg/m^2^, on day 1, cisplatin, 60 mg/m^2^, on day 1, fluorouracil, 200 mg/m^2^, 21-d continuous infusion, 3 postoperative cycles (n = 60)
Zhao et al,^41^ 2015	2012-2013, China	Adenocarcinoma	Group A: preoperative simultaneous CRT, 45 Gy, oxaliplatin, 130 mg/m^2^, on day 1, capecitabine, 2000 mg/m^2^, on days 1-14 (n = 36); group B: surgery alone (n = 40)

Abbreviations: ACCORD, Actions Concertées dans les Cancers Colo-Rectaux et Digestifs; AEG, adenocarcinoma of the esophagus or esophageal junction; AGITG, Australasian Gastro-Intestinal Trials Group; CALGB, Cancer and Leukemia Group B; CROSS, Chemoradiotherapy for Oesophageal Cancer Followed by Surgery Study; CRT, chemoradiotherapy; EORTC, European Organisation for Research and Treatment of Cancer; FFCD, Federation Francophone de Cancerologie Digestive; IPD, individual patient data; MAGIC, Medical Research Council Adjuvant Gastric Infusional Chemotherapy; NeoRes, Neoadjuvant Chemotherapy Versus Radiochemotherapy for Cancer of the Esophagus or Cardia; POET, Preoperative Therapy in Esophagogastric Adenocarcinoma Trial; RTOG, Radiation Therapy Oncology Group; TROG, Trans Tasman Radiation Oncology Group.

### Risk of Bias and Data Quality

Overall risk of bias was low in 12 trials^4,8,9,10,11,12,15,19,36,37,38,39^ and moderate in 5 trials^14,16,35,40,41^ (eFigures 2 and 3 in Supplement 1). Comparison-adjusted funnel plots for each outcome showed no small-study effect (eFigure 4 in Supplement 1). No implausible outliers were identified. Any differences between IPD and published results were small (eResults in Supplement 1).

### Associations of Treatments With Outcomes

**Figure 2.**
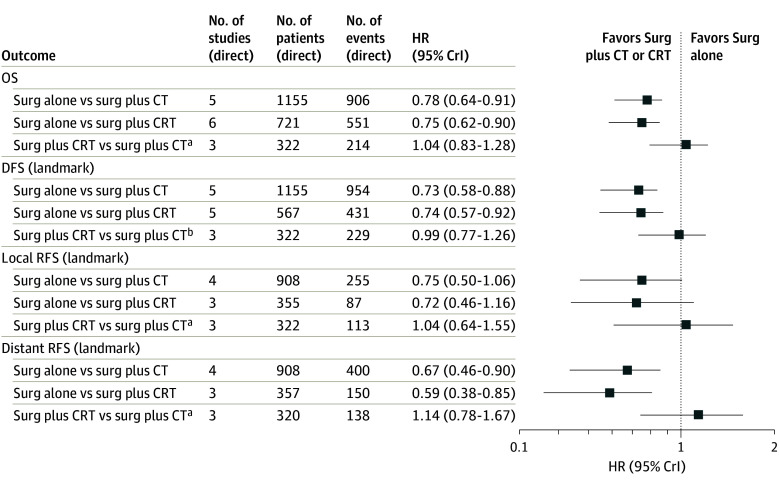
Summary Forest Plot of Survival Outcomes The plot displays the network estimates of the age- and sex-adjusted hazard ratios (HRs) and the 95% credible interval (CrI) for each survival outcome. The numbers of studies, patients, and events are related to the direct comparison through the HR and 95% CrI, estimated from the network using direct and indirect evidence for each outcome. CRT indicates chemoradiotherapy; CT, chemotherapy; DFS, disease-free survival; OS, overall survival; RFS, recurrence-free survival; and surg, surgery. ^a^Favors surgery plus CRT. ^b^Favors surgery plus CT.

Associations of treatments with outcomes were first assessed by calculating network estimates of the age- and sex-adjusted HRs. A corresponding summary forest plot for the survival outcomes is shown in Figure 2. Overall survival results favored preoperative and/or perioperative chemotherapy plus surgery (HR, 0.78 [95% CrI, 0.64-0.91]; 3-year difference, 90 deaths per 1000 patients) and preoperative CRT plus surgery (HR, 0.75 [95% CrI, 0.62-0.90]; 3-year difference: 105 deaths per 1000 patients) over surgery alone. The 2-stage bayesian NMA estimated an HR of 1.04 (95% CrI, 0.83-1.28; 3-year difference, 15 deaths per 1000 patients) in favor of preoperative CRT plus surgery vs preoperative and/or perioperative chemotherapy plus surgery with low between-study heterogeneity (τ = 0.12). For DFS, results favored preoperative and/or perioperative chemotherapy plus surgery over surgery (HR, 0.73 [95% CrI, 0.58-0.88]) and preoperative CRT plus surgery over surgery (HR, 0.74 [95% CrI, 0.57-0.92]). The 2-stage bayesian network estimated an HR of 0.99 (95% CrI, 0.77-1.26) for preoperative and/or perioperative chemotherapy plus surgery vs preoperative CRT plus surgery with low heterogeneity (τ = 0.17). Both preoperative and/or perioperative chemotherapy plus surgery (HR, 0.67 [95% CrI, 0.46-0.90]) and preoperative CRT plus surgery (HR, 0.59 [95% CrI, 0.38-0.85]) were associated with longer distant RFS compared with surgery alone. For local RFS, 95% CrIs included 1.

**Figure 3.**
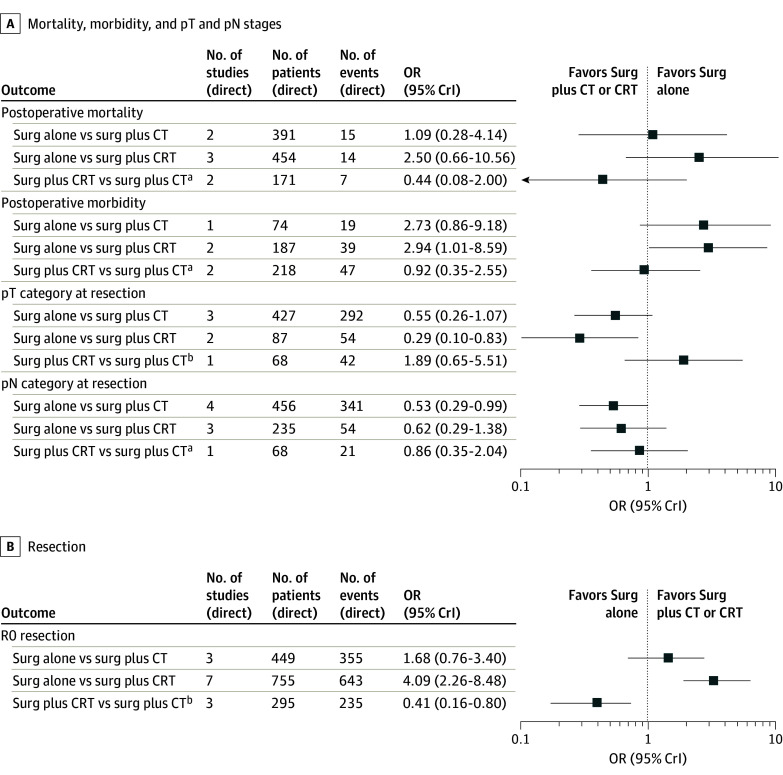
Summary Forest Plot of Binary Outcomes The plot displays the network estimates of the odds ratios (ORs) and the 95% credible interval (CrI) for each binary outcome. The numbers of studies, patients, and events are related to the direct comparison through the OR and 95% CrI, estimated from the network using direct and indirect evidence for each outcome. CRT indicates chemoradiotherapy; CT, chemotherapy; and surg, surgery. ^a^Favors surgery plus CT. ^b^Favors surgery plus CRT.

Results for the binary outcomes are shown in Figure 3. Postoperative mortality was not significantly more frequent after CRT plus surgery compared with surgery alone (OR, 2.50 [95% CrI, 0.66-10.56]) or after chemotherapy plus surgery compared with CRT plus surgery (OR, 0.44 [95% CrI, 0.08-2.00]). Postoperative morbidity was significantly higher after preoperative CRT compared with surgery alone (OR, 2.94 [95% CrI, 1.01-8.59]). The pT category was lower for patients after preoperative CRT plus surgery compared with surgery alone (OR, 0.29 [95% CrI, 0.10-0.83]), while the pN category was lower after preoperative chemotherapy plus surgery compared with surgery alone (OR, 0.53 [95% CrI, 0.29-0.99]). R0 resection was more frequent after preoperative CRT than surgery alone (OR, 4.09 [95% CrI, 2.26-8.48]) and less frequent after preoperative chemotherapy than preoperative CRT (OR, 0.41 [95% CrI, 0.16-0.80]).

Subsequently, a comparison of direct and indirect estimates was made. It showed no inconsistencies for any outcome and largely matched the network estimates (eFigure 5 in Supplement 1). The certainty of the evidence according to the Grading of Recommendations Assessment, Development and Evaluation system was high for OS, DFS, and RFS and moderate for morbidity and mortality.

Last, modalities were ranked by calculating SUCRA scores and median ranks (Table 2). For all survival outcomes, R0 resection, and pT category, preoperative CRT plus surgery had the highest and surgery alone had the lowest probability of being the best treatment. For pN category, preoperative and/or perioperative chemotherapy plus surgery had the highest and surgery alone had the lowest probability of being the best treatment. For postoperative morbidity and mortality, surgery alone had the highest and preoperative CRT plus surgery had the lowest probability of being the best treatment.

**Table 2.** Surface Under the Cumulative Ranking Scores for All Outcomes

Outcome	Score by treatment group (median rank [95% CrI])^a^		
	Surgery plus CRT	Surgery plus chemotherapy	Surgery alone
OS	0.82 (1 [1-2])	0.68 (2 [1-2])	<0.01 (3 [3-3])
DFS	0.72 (2 [1-2])	0.78 (1 [1-2])	<0.01 (3 [3-3])
Local RFS	0.75 (1 [1-3])	0.69 (2 [1-3])	0.06 (3 [2-3])
Distant RFS	0.88 (1 [1-2])	0.61 (2 [1-2])	0.01 (3 [3-3])
Postoperative mortality	0.10 (3 [1-3])	0.66 (2 [1-3])	0.74 (1 [1-3])
Postoperative morbidity	0.22 (3 [2-3])	0.31 (2 [1-3])	0.97 (1 [1-2])
R0 resectability	1.00 (1 [1-1])	0.47 (2 [2-3])	0.04 (3 [2-3])
pT category on resection	0.94 (1 [1-2])	0.54 (2 [1-3])	0.02 (3 [2-3])
pN category on resection	0.62 (2 [1-3])	0.82 (1 [1-2])	0.06 (3 [2-3])

Abbreviations: CrI, credible interval; CRT, chemoradiotherapy; DFS, disease-free survival; OS, overall survival; RFS, recurrence-free survival.

^a^ A value of 1.00 indicates a treatment certain to be the best; 0, certain to be the worst.

A single study assessed pCR in both treatment groups with only 5 events (1 of 36 after chemotherapy and 4 of 35 after CRT).^36^ As the MAGIC (Medical Research Council Adjuvant Gastric Infusional Chemotherapy) study^9^ and the study by Tian et al^39^ compared chemotherapy plus surgery with surgery, a relative treatment effect could not be calculated. Thirteen of 130 participants in these studies achieved pCR.

Toxicity and quality of life were assessed descriptively given that no meta-analysis was possible. Five studies^4,10,12,37,41^ reported toxicity results. In all these, toxicity was assessed in only 1 treatment arm, because the comparator was surgery alone. Individual study results are reported in the eTable in Supplement 1. A total of 227 of 402 participants (56.4%) experienced toxic effects of any grade. Quality of life was reported by 4 trials^4,14,16,35^ with IPD available only from one.^4^

### Subgroup and Sensitivity Analyses

A summary forest plot with the network estimates of the subgroup analyses for OS is shown in eFigure 6 in Supplement 1. A forest plot with NMA results of the subgroup analyses for DFS is shown in eFigure 7 in Supplement 1. The estimates resemble those of the analyses of the overall study populations for most subgroups, with wider 95% CrIs due to fewer patients in the respective subgroups. Sensitivity analyses for all outcomes suggested robustness of the models with respect to the choice of the priors (eFigure 8 in Supplement 1).

## Discussion

This IPD NMA compared preoperative CRT plus surgery, preoperative and/or perioperative chemotherapy plus surgery, and surgery alone in 2549 patients with AEG from 17 RCTs. Risk of bias was low in most studies and moderate in the remainder of the trials, with selective reporting being the most frequent reason for moderate risk. While treatment adherence was high for preoperative CRT and chemotherapy, it was considerably lower for postoperative chemotherapy.

The NMA shows that both preoperative CRT plus surgery and preoperative and/or perioperative chemotherapy plus surgery are associated with longer OS, DFS, and distant RFS compared with surgery alone. The association with OS was consistent throughout most subgroups. In some subgroups, results were inconclusive, probably due to lower statistical power. The comparisons between preoperative and/or perioperative chemotherapy plus surgery and preoperative CRT plus surgery showed no differences regarding survival. Following surgery after either modality, pT and pN categories were lower than following surgery alone, which reflects downstaging by preoperative treatments.^42^ R0 resection was more frequent after preoperative treatment than upfront surgery, but the difference was more pronounced after CRT than chemotherapy. Downstaging and R0 resection are associated with survival^42,43^; therefore, these findings constitute a relationship between the treatments and their survival outcome. Pathological complete response, which is another surrogate,^44,45^ was only assessed in 3 RCTs^9,36,39^ and could therefore not be validly analyzed. Both preoperative modalities appeared to have higher postoperative morbidity compared with surgery alone, although there was an association only for preoperative CRT. Postoperative mortality was higher after CRT than after chemotherapy or surgery alone, but results were not statistically significant. Overall, the risk of postoperative complications was slightly elevated after preoperative treatment and especially CRT. Possible mechanisms comprise immunosuppression and tissue vulnerability,^46^ and special attention is warranted to prevent, detect, and treat complications early in patients who underwent preoperative therapy.

A recently updated IPD NMA chose a different approach and included patients with esophageal carcinoma regardless of histology.^47^ The included trials overlapped with ours, and survival results were strikingly similar. Of note, histology was not identified as an effect modifier. Previous meta-analyses included both patients with AEG and esophageal squamous cell carcinoma,^5,48,49^ only 1 of the 2 modalities preoperative chemotherapy or CRT,^50^ or studies using chemotherapy as well as those using CRT without being able to compare the 2 modalities by integrating direct and indirect evidence in an NMA.^7,13,51^ In line with our results, they consistently showed the advantage of preoperative CRT plus surgery or preoperative and/or perioperative chemotherapy plus surgery over surgery alone with regard to survival and surrogate outcomes like downstaging and complete resection.^5,7,13,47,48,50^ Postoperative morbidity and mortality were also assessed, and no relevant differences between the modalities were found. One meta-analysis^51^ exclusively assessed safety and found no differences between preoperative therapy plus surgery and surgery alone but did not include data pertaining only to patients with AEG. These findings are different from ours, which showed safety concerns after preoperative CRT.

Our analyses could not demonstrate a clear survival advantage for 1 of the 2 multimodal approaches. After our search, results of the Neo-AEGIS trial, which compared preoperative CRT with perioperative chemotherapy, were published.^17^ It closed prematurely following futility analyses, and no survival or mortality differences were found, which is in line with our findings. Ongoing RCTs comparing the 2 approaches will further add to the evidence.^18,19,20^

The observation that preoperative treatment is completed by a much higher proportion of patients than the postoperative part of perioperative treatment is in agreement with previous evidence.^6^ It probably reflects both the fact that more patients are unable to initiate chemotherapy following extensive surgery and that toxicity is more often treatment limiting in the postoperative setting. It underscores the importance of administering a sufficient dose of preoperative chemotherapy. A possible incremental benefit of postoperative continuation of chemotherapy could not be assessed in our analyses.

The included RCTs were conducted over a wide time range and included various treatments, which makes it difficult to discern treatment effects of specific drug combinations. The duration of preoperative chemotherapy was heterogenous and might have modulated single-trial effects. The notion that longer chemotherapy is associated with longer survival was not supported by the OE05 trial (United Kingdom Medical Research Council esophageal cancer trial),^52^ which compared 2 and 4 cycles of preoperative chemotherapy with no survival difference, and by another RCT^53^ that showed no survival difference for CRT with or without induction chemotherapy. Although perioperative chemotherapy usually consists of longer and more dose-intense chemotherapy than CRT, this does not translate into longer survival.

Recently, checkpoint inhibitors have become standard of care in certain treatment lines for many solid tumors. Besides, *ERBB2* (formerly *HER2*) blockade is established for overexpressing AEG in metastatic settings. These treatments were not in the scope of our analyses. Surrogate end point results from RCTs on preoperative checkpoint inhibition are promising.^54,55,56^ In 1 trial,^56^ however, this did not translate into longer event-free survival or OS, while other trials are still to report survival.^54,55^ Perioperative anti-*ERBB2* treatment has shown benefits for pCR.^57^ Notwithstanding, in a phase 3 trial, the addition of *ERBB2* blockade to preoperative CRT did not prolong survival, which questions pCR as a surrogate marker.^58^

### Limitations

This meta-analysis has limitations. By combining data from trials with different inclusion and exclusion criteria and treatments, heterogeneity was inevitable. While internal validity was lower than in a single RCT, external validity was higher. In light of the sensitive search strategy in multiple databases and reference lists and the possible inclusion of non–English language publications, we are confident that no relevant trials were missed. An IPD NMA allowed for a valid comparison of all 3 modalities. This distinguishes our NMA from previous meta-analyses that had to rely on AD, could not perform specific analyses for AEG and other subgroups, and were thus prone to bias.^5,49^ No evidence of inconsistency of the NMA was found in a node-splitting model,^30^ in which each treatment comparison was split into direct and indirect evidence. The sensitivity analyses revealed robust results with regard to the priors for the bayesian NMA models. For few comparisons of binary end points, where the 95% CrI barely excluded or included 1, the choice of prior changed the significance of the pooled effect. Therefore, more studies would reduce the influence of the prior and enhance the certainty of the results. Some included trials consisted of patients with AEG and squamous cell carcinoma. Thanks to IPD or stratified AD, we included only patients with AEG, rendering results specific to that population. Inclusion criteria of single RCTs regarding tumor location, stage, and resectability were strict, thus minimizing the likelihood that patients who had gastric, metastatic, or irresectable tumors were included. In the single trials, different treatments were used. This made it difficult to apply the results to all existing preoperative regimens or to recommend a specific regimen. Although this IPD NMA includes all available evidence from RCTs published during the search period, the overall number of included trials and thus patients, specifically in some subgroups, is still limited, requiring more evidence for some comparisons to be able to draw definite conclusions.

## Conclusions

Findings of this IPD NMA suggest that both preoperative CRT plus surgery and preoperative and/or perioperative chemotherapy plus surgery are associated with longer survival of patients with AEG compared with surgery alone. No differences between the effect of the 2 modalities could be found. The association might be mediated through tumor downstaging and a higher probability of complete resection. Future research should focus on identifying specific groups of patients in whom 1 of the 2 modalities could be more effective, and on the integration of checkpoint inhibitors and targeted therapies into preoperative treatment schemes.

## Notes

Supplement 1. **eAppendix 1.** Predefined Search Strategy **eAppendix 2.** Data Quality Checks **eAppendix 3.** Assessment of Risk of Bias in Included Studies **eAppendix 4.** Variables, Eligibility, and Missing Data **eAppendix 5.** Statistical Methods **eAppendix 6.** Subgroup Analyses and Certainty Assessment **eResults.** Excluded Studies, Differences Between IPD Datasets and Published Results, and Sensitivity Analyses **eFigure 1.** Network Graphs for Survival Outcomes and Binary Outcomes **eFigure 2.** Risk of Bias per Domain and Trials **eFigure 3.** Risk of Bias Summary Across Trials **eFigure 4.** Comparison-Adjusted Funnel Plots of Each Outcome **eTable.** Frequency of Toxicity Events (Any Grade) in the Single Trials Reporting This Outcome **eFigure 5.** Assessment of Inconsistency by the Node-Splitting Approach **eFigure 6.** NMA Results of the Subgroup Analyses for OS **eFigure 7.** NMA Results of the Subgroup Analyses for DFS **eFigure 8.** Results of the Sensitivity Analyses
eReferences.

Supplement 2.
Data Sharing Statement
