# Errors in Figure 3

**DOI:** 10.1001/jamanetworkopen.2025.35510

**Published:** 2025-09-11

In the Original Investigation titled “Preoperative Chemoradiotherapy vs Chemotherapy for Adenocarcinoma of the Esophagogastric Junction: A Network Meta-Analysis,”^1^ published on August 2, 2024, there were errors in Figure 3. The R0 resection outcome should appear as a separate panel, and footnote b should be cited instead of footnote a for the surg plus CRT vs surg plus CT comparison. This article has been corrected.
