# Supplementary material for: Preoperative Chemoradiotherapy vs Chemotherapy for Adenocarcinoma of the Esophagogastric Junction: A Network Meta-Analysis
Source: JAMA Netw Open. 2024 Aug 2;7(8):e2425581. doi: 10.1001/jamanetworkopen.2024.25581 (PMC11297377; doi:10.1001/jamanetworkopen.2024.25581)
Supplement: Supplement 1. — eAppendix 1. Predefined Search Strategy eAppendix 2. Data Quality Checks eAppendix 3. Assessment of Risk of Bias in Included Studies eAppendix 4. Variables, Eligibility, and Missing Data eAppendix 5. Statistical Methods eAppendix 6. Subgroup Analyses and Certainty Assessment eResults. Excluded Studies, Differences Between IPD Datasets and Published Results, and Sensitivity Analyses eFigure 1. Network Graphs for Survival Outcomes and Binary Outcomes eFigure 2. Risk of Bias per Domain and Trials eFigure 3. Risk of Bias Summary Across Trials eFigure 4. Comparison-Adjusted Funnel Plots of Each Outcome eTable. Frequency of Toxicity Events (Any Grade) in the Single Trials Reporting This Outcome eFigure 5. Assessment of Inconsistency by the Node-Splitting Approach eFigure 6. NMA Results of the Subgroup Analyses for OS eFigure 7. NMA Results of the Subgroup Analyses for DFS eFigure 8. Results of the Sensitivity Analyses eReferences. [file jamanetwopen-e2425581-s001.pdf]

Supplementary Online Content

Ronellenfitsch U, Friedrichs J, Barbier E, et al. Preoperative chemoradiotherapy vs chemotherapy for adenocarcinoma of the esophagogastric junction: a network meta-analysis. *JAMA Netw Open*. 2024;7(8):e2425581. doi:10.1001/jamanetworkopen.2024.25581

eAppendix 1. Predefined Search Strategy
eAppendix 2. Data Quality Checks
eAppendix 3. Assessment of Risk of Bias in Included Studies
eAppendix 4. Variables, Eligibility, and Missing Data
eAppendix 5. Statistical Methods
eAppendix 6. Subgroup Analyses and Certainty Assessment
eResults. Excluded Studies, Differences Between IPD Datasets and Published Results, and Sensitivity Analyses
eFigure 1. Network Graphs for Survival Outcomes and Binary Outcomes
eFigure 2. Risk of Bias per Domain and Trials
eFigure 3. Risk of Bias Summary Across Trials
eFigure 4. Comparison-Adjusted Funnel Plots of Each Outcome
eTable. Frequency of Toxicity Events (Any Grade) in the Single Trials Reporting This Outcome
eFigure 5. Assessment of Inconsistency by the Node-Splitting Approach
eFigure 6. NMA Results of the Subgroup Analyses for OS
eFigure 7. NMA Results of the Subgroup Analyses for DFS
eFigure 8. Results of the Sensitivity Analyses
eReferences.

This supplementary material has been provided by the authors to give readers additional information about their work.

© 2024 Ronellenfitsch U et al. *JAMA Network Open*.

eAppendix 1. Predefined Search Strategy

PubMed

1 "adenocarcinoma Of esophagus"[Supplementary Concept] OR "Esophageal neoplasms"[MeSH Terms] OR "Barrett Esophagus"[MeSH Terms] OR "esophagus neoplas*"[Text Word] OR "esophagus tumor*"[Text Word] OR "esophagus tumour*"[Text Word] OR "esophagus cancer*"[Text Word] OR "esophagus carcinoma*"[Text Word] OR "esophagus adenocarcinoma*"[Text Word] OR "neoplasm of the esophagus"[Text Word] OR "neoplasm of esophagus"[Text Word] OR "tumor of the esophagus"[Text Word] OR "tumor of esophagus" [Text Word] OR "tumour of the esophagus"[Text Word] OR "cancer of the esophagus"[Text Word] OR "cancer of esophagus"[Text Word] OR "carcinoma of the esophagus"[Text Word] OR "carcinoma of esophagus"[Text Word] OR "adenocarcinoma of the esophagus"[Text Word] OR "adenocarcinoma Of esophagus"[Text Word] OR "esophageal neoplas*"[Text Word] OR "esophageal tumor*"[Text Word] OR "esophageal tumour*"[Text Word] OR "esophageal cancer*"[Text Word] OR "esophageal carcinoma*"[Text Word] OR "esophageal malignancy"[Text Word] OR "esophageal adenocarcinoma*"[Text Word] OR "esophageal adenoma*"[Text Word] OR "oesophagus neoplas*"[Text Word] OR "oesophagus tumor*"[Text Word] OR "oesophagus tumour*"[Text Word] OR "oesophagus cancer*"[Text Word] OR "oesophagus carcinoma*"[Text Word] OR "oesophagus adenocarcinoma*"[Text Word] OR "neoplasm of the oesophagus"[Text Word] OR "tumor of the oesophagus"[Text Word] OR "tumour of the oesophagus"[Text Word] OR "tumour of oesophagus"[Text Word] OR "cancer of the oesophagus"[Text Word] OR "cancer of oesophagus"[Text Word] OR "carcinoma of the oesophagus"[Text Word] OR "carcinoma of oesophagus"[Text Word] OR "adenocarcinoma of oesophagus"[Text Word] OR "oesophageal neoplas*"[Text Word] OR "oesophageal tumor*"[Text Word] OR "oesophageal tumour*"[Text Word] OR "oesophageal cancer*"[Text Word] OR "oesophageal carcinoma*"[Text Word] OR "oesophageal malignancy"[Text Word] OR "oesophageal adenocarcinoma*"[Text Word] OR "barrett neoplas*"[Text Word] OR "barrett cancer*"[Text Word] OR "barrett carcinoma*"[Text Word] OR "barrett adenocarcinoma*"[Text Word] OR "barrett s neoplas*"[Text Word] OR "barrett s tumor*"[Text Word] OR "barrett s tumour*" [Text Word] OR "barrett s cancer*"[Text Word] OR "barrett s carcinoma*"[Text Word] OR "barrett s adenocarcinoma*"[Text Word] OR "barrett s neoplas*"[Text Word] OR "barrett s tumor*"[Text Word] OR "barrett s tumour*"[Text Word] OR "barrett s cancer*"[Text Word] OR "barrett s carcinoma*"[Text Word] OR "barrett s adenocarcinoma*"[Text Word] OR "cardia tumor*"[Text Word] OR "cardia tumour*"[Text Word] OR "cardia cancer*"[Text Word] OR "cardia carcinoma*"[Text Word] OR "cardia adenocarcinoma*"[Text Word]
2 "esophageal Squamous Cell carcinoma"[MeSH Terms] OR "esophagus squamous cell carcinoma*"[Text Word] OR "oesophagus squamous cell carcinoma*"[Text Word] OR "esophageal squamous cell carcinoma*"[Text Word] OR "oesophageal squamous cell carcinoma*"[Text Word]
3 ("Esophagogastric Junction"[MeSH Terms] OR "gastroesophageal junction*"[Text Word] OR "gastrooesophageal junction*"[Text Word] OR "esophagogastric junction*"[Text Word] OR "oesophagogastric junction*"[Text Word]) AND ("adenocarcinoma"[MeSH Terms] OR "neoplas*"[Text Word] OR "tumor*"[Text Word] OR "tumour*"[Text Word] OR "cancer*"[Text Word] OR "carcinoma*"[Text Word] OR "malignancy"[Text Word] OR "adenocarcinoma*"[Text Word] OR "adenoma*"[Text Word])
4 "Chemoradiotherapy"[MeSH Terms] OR "radiotherapy, adjuvant"[MeSH Terms] OR "adjuvant radio*"[Text Word] OR "chemoradiotherap*"[Text Word] OR "chemoradio therap*"[Text Word] OR "radiochemotherap*"[Text Word] OR "radio chemotherap*" [Text Word] OR "chemoradiation*"[Text Word] OR "chemo radiation*"[Text Word]
5 "Induction Chemotherapy"[MeSH Terms] OR "chemotherapy, adjuvant"[MeSH Terms] OR "Neoadjuvant Therapy"[MeSH Terms] OR "Antineoplastic Agents"[MeSH Terms] OR "Antineoplastic Combined Chemotherapy Protocols"[MeSH Terms] OR "Combined Modality Therapy"[MeSH Terms:noexp] OR "chemotherap*"[Text Word] OR "chemo therap*"[Text Word] OR "adjuvant chemo*"[Text Word] OR "antineoplastic drug combination*"[Text Word] OR "antitumor drug combination*"[Text Word] OR "anticancer agent combination*"[Text Word] OR "anticancer drug combination*"[Text Word] OR "anticancer drugs combination*"[Text Word] OR "combined anticanceragent*"[Text Word] OR "combined anticancer drug*"[Text Word]
6 "General Surgery"[MeSH Terms] OR "Surgical Oncology"[MeSH Terms] OR "Digestive System Surgical Procedures"[MeSH Terms] OR "esophagectom*"[Text Word] OR "oesophagectom*"[Text Word] OR "esophago gastrectom*"[Text Word] OR "oesophagogastrectom*"[Text Word] OR "surg*"[Text Word] OR "operat*"[Text Word] OR "preoperative*"[Text Word]
7 "clinical trials as topic"[MeSH Terms:noexp] OR "randomized controlled trial"[Publication Type] OR "controlled clinical trial"[Publication Type] OR "randomized"[Title/Abstract] OR "placebo"[Title/Abstract] OR "randomly"[Title/Abstract] OR "trial"[Title]
8 1 OR 2 OR 3

9 4 OR 5
10 6 AND 7 AND 8 AND 9

Cochrane Library

1 [mh "Esophageal neoplasms"] OR [mh "Barrett Esophagus"] OR esophag* NEAR/2 neoplas*:ti,ab,kw OR esophag* NEAR/2 tumor*:ti,ab,kw OR esophag* NEAR/2 tumour*:ti,ab,kw OR esophag* NEAR/2 cancer*:ti,ab,kw OR esophag* NEAR/2 carcinoma*:ti,ab,kw OR esophag* NEAR/2 malignancy:ti,ab,kw OR esophag* NEAR/2 adenocarcinoma*:ti,ab,kw OR esophag* NEAR/2 adenoma*:ti,ab,kw ORoesophag* NEAR/2 neoplas*:ti,ab,kw OR oesophag* NEAR/2 tumor*:ti,ab,kw OR oesophag* NEAR/2 tumour*:ti,ab,kw OR oesophag* NEAR/2 cancer*:ti,ab,kw OR oesophag* NEAR/2 carcinoma*:ti,ab,kw OR oesophag* NEAR/2 malignancy:ti,ab,kw OR oesophag* NEAR/2 adenocarcinoma*:ti,ab,kw OR oesophag* NEAR/2 adenoma*:ti,ab,kw ORBarrett* NEAR/2 neoplas*:ti,ab,kw OR Barrett* NEAR/2 tumor*:ti,ab,kw OR Barrett* NEAR/2 tumour*:ti,ab,kw OR Barrett* NEAR/2 cancer*:ti,ab,kw OR Barrett* NEAR/2 carcinoma*:ti,ab,kw OR Barrett* NEAR/2 malignancy:ti,ab,kw OR Barrett* NEAR/2 adenocarcinoma*:ti,ab,kw OR Barrett* NEAR/2 adenoma*:ti,ab,kw ORcardia NEAR/2 neoplas*:ti,ab,kw OR cardia NEAR/2 tumor*:ti,ab,kw OR cardia NEAR/2 tumour*:ti,ab,kw OR cardia NEAR/2 cancer*:ti,ab,kw OR cardia NEAR/2 carcinoma*:ti,ab,kw OR cardia NEAR/2 malignancy:ti,ab,kw OR cardia NEAR/2 adenocarcinoma*:ti,ab,kw OR cardia NEAR/2 adenoma*:ti,ab,kw
2 [mh "esophageal Squamous Cell carcinoma"] OR esophag* NEAR/2 Squamous NEAR/2 Cell NEAR/2 carcinoma*:ti,ab,kw OR oesophag* NEAR/2 Squamous NEAR/2 Cell NEAR/2 carcinoma*:ti,ab,kw
3 ([mh "Esophagogastric Junction"] OR "gastroesophageal Junction*":ti,ab,kw OR "gastrooesophageal Junction*":ti,ab,kw OR "esophagogastric Junction*":ti,ab,kw OR "oesophagogastric Junction*":ti,ab,kw) AND ([mh "adenocarcinoma"] OR "neoplas*":ti,ab,kw OR "tumor*":ti,ab,kw OR "tumour*":ti,ab,kw OR "cancer*":ti,ab,kw OR "carcinoma*":ti,ab,kw OR "malignancy":ti,ab,kw OR "adenocarcinoma*":ti,ab,kw OR "adenoma*":ti,ab,kw)
4 [mh "Chemoradiotherapy"] OR [mh "Radiotherapy, Adjuvant"] OR "Adjuvant Radio*":ti,ab,kw OR chemoradiotherap*:ti,ab,kw OR chemoradio NEAR/2 therap*:ti,ab,kw OR radiochemotherap*:ti,ab,kw OR radio NEAR/2 chemotherap*:ti,ab,kw OR chemoradiation*:ti,ab,kw OR chemo NEAR/2 radiation*:ti,ab,kw OR chemoradiation*:ti,ab,kw OR "chemo radiation*":ti,ab,kw **5** [mh "Induction Chemotherapy"] OR [mh "Chemotherapy, Adjuvant"] OR [mh "Neoadjuvant Therapy"] OR [mh "Antineoplastic Agents"] OR [mh "Antineoplastic Combined Chemotherapy Protocols"] OR [mh ^"Combined Modality Therapy"] ORchemotherap*:ti,ab,kw OR chemo NEAR/2 therap*:ti,ab,kw OR Adjuvant NEAR/2 Chemo:ti,ab,kw OR antineoplastic NEAR/2 drug NEAR/2 combination*:ti,ab,kw OR antitumor NEAR/2 drug NEAR/2 combination*:ti,ab,kw OR anticancer NEAR/2 agent NEAR/2 combination*:ti,ab,kw OR anticancer NEAR/2 drug NEAR/2 combination*:ti,ab,kw OR anticancer NEAR/2 drugs NEAR/2 combination*:ti,ab,kw OR combined NEAR/2 anticancer NEAR/2 agent*:ti,ab,kw OR combined NEAR/2 anticancer NEAR/2 drug*:ti,ab,kw
6 [mh "General Surgery"] OR [mh "Surgical Oncology"] OR[mh "Digestive System Surgical Procedures"] OR Esophagectom*:ti,ab,kw OROesophagectom*:ti,ab,kw ORPesophag* NEAR/2 gastrectom*:ti,ab,kw ORoesophag* NEAR/2 gastrectom*:ti,ab,kw ORsurg*:ti,ab,kw OR operat*:ti,ab,kw OR preoperative*:ti,ab,kw
7 1 OR 2 OR 3
8 4 OR 5
9 6 AND 7 AND 8

CINAHL

1 (MH "Esophageal Neoplasms+") OR (MH "Barrett Esophagus") OR TX (esophag* N5 neoplas* OR esophag* N5 tumor* OR esophag* N5 tumour* OR esophag* N5 cancer* OR esophag* N5 carcinoma* OR esophag* N5 malignancy OR esophag* N5 adenocarcinoma* OR esophag* N5 adenoma* OR neoplas* N5 esophagus OR tumor* N5 esophagus OR tumour* N5 esophagus OR cancer* N5 esophagus OR carcinoma* N5 esophagus OR malignancy N5 esophagus OR adenocarcinoma* N5 esophagus OR adenoma* N5 esophagus OR oesophag* N5 neoplas* OR oesophag* N5 tumor* OR oesophag* N5 tumour* OR oesophag* N5 cancer* OR oesophag* N5 carcinoma* OR oesophag* N5 malignancy OR oesophag* N5 adenocarcinoma* OR oesophag* N5 adenoma* OR neoplas* N5 oesophagus OR tumor* N5 oesophagus OR tumour* N5 oesophagus OR cancer* N5 oesophagus OR carcinoma* N5 oesophagus OR malignancy* N5 oesophagus OR adenocarcinoma* N5 oesophagus OR adenoma* N5 oesophagus OR barrett* N5 neoplas* OR barrett* N5 tumor* OR barrett* N5 tumour* OR barrett* N5 cancer* OR barrett* N5 carcinoma* OR barrett* N5 malignancy OR barrett* N5 adenocarcinoma* OR barrett* N5 adenoma* OR cardia N5 neoplas* OR cardia N5 tumor* OR cardia N5

tumour* OR cardia N5 cancer* OR cardia N5 carcinoma* OR cardia N5 malignancy OR cardia N5 adenocarcinoma* OR cardia N5 adenoma*)
2 (TX (esophag* N5 Squamous N5 Cell N5 carcinoma* OR oesophag* N5 Squamous N5 Cell N5 carcinoma*))
3 (TX (gastroesophageal N5 Junction* OR gastrooesophageal N5 Junction* OR esophagogastric N5 Junction* OR oesophagogastric N5 Junction*)) AND (MH "Adenocarcinoma" OR (TX (neoplas* OR tumor* OR tumour* OR cancer* OR carcinoma* OR malignancy OR adenocarcinoma* OR adenoma*)))
4 (MH "Chemoradiotherapy+") OR (MH "Radiotherapy, Adjuvant+") OR TX (chemoradiotherap* OR Adjuvant N5 Radio* OR chemoradio N5 therap* OR radiochemotherap* OR radio N5 chemotherap* OR chemoradiation* OR chemo N5 radiation* OR chemoradiation* OR chemo N5 radiation*)
5 (MH "Chemotherapy, Cancer+") OR (MH "Neoadjuvant Therapy") OR (MH "Antineoplastic Agents+") OR (MH "Combined Modality Therapy+") OR TX (chemotherap* OR chemo N5 therap* OR adjuvant N5 chemo* OR antineoplastic N5 drug N5 combination* OR antitumor N5 drug N5 combination* OR anticancer N5 agent N5 combination* OR anticancer N5 drug N5 combination* OR anticancer N5 drugs N5 combination* OR combined N5 anticancer N5 agent* OR combined N5 anticancer N5 drug*)
6 (MH "Surgery, Operative") OR (MH "Surgery, Operative+") OR TX (surg*" OR operat* OR preoperative* Esophagectom* OR Oesophagectom* OR esophag* N5 gastrectom* OR oesophag* N5 gastrectom*)
7 MH randomized controlled trials OR MH double-blind studies OR MH single-blind studies OR MH random assignment OR MH pretestposttest design OR MH cluster sample OR TI (randomised OR randomized) OR AB (random*) OR TI (trial) OR (MH (sample size) AND AB (assigned OR allocated OR control)) OR MH (placebos) OR PT (randomized controlled trial) OR AB (control W5 group) OR MH (crossover design) OR MH (comparative studies) OR AB (cluster W3 RCT)
8 1 OR 2 OR 3
9 4 OR 5
10 6 AND 7 AND 8 AND 9

ClinicalTrials.gov

1 EXPAND[Concept] ("esophagus neoplasm" OR "Barrett Esophagus" OR "esophagus tumor" OR "esophagus tumour" OR "esophagus cancer" OR "esophagus carcinoma" OR "esophagus malignancy" OR "esophagus adenocarcinoma" OR "esophagus adenoma" OR "neoplasm of the esophagus" OR "neoplasm of esophagus" OR "tumor of the esophagus" OR "tumor of esophagus" OR "tumour of the esophagus" OR "tumour of esophagus" OR "cancer of the esophagus" OR "cancer of esophagus" OR "carcinoma of the esophagus" OR "carcinoma of esophagus" OR "malignancy of the esophagus" OR "malignancy of esophagus" OR "adenocarcinoma of the esophagus" OR "adenocarcinoma of esophagus" OR "adenoma of the esophagus" OR "adenoma of esophagus" OR "esophageal neoplasm" OR "esophageal tumor" OR "esophageal tumour" OR "esophageal cancer" OR "esophageal carcinoma" OR "esophageal malignancy" OR "esophageal adenocarcinoma" OR "esophageal adenoma" OR "oesophagus neoplasm" OR "oesophagus tumor" OR "oesophagus tumour" OR "oesophagus cancer" OR "oesophagus carcinoma" OR "oesophagus malignancy" OR "oesophagus adenocarcinoma" OR "oesophagus adenoma" OR "neoplasm of the oesophagus" OR "neoplasm of oesophagus" OR "tumor of the oesophagus" OR "tumor of oesophagus" OR "tumour of the oesophagus" OR "tumour of oesophagus" OR "cancer of the oesophagus" OR "cancer of oesophagus" OR "carcinoma of the oesophagus" OR "carcinoma of oesophagus" OR "malignancy of the oesophagus" OR "malignancy of oesophagus" OR "adenocarcinoma of the oesophagus" OR "adenocarcinoma of oesophagus" OR "adenoma of the oesophagus" OR "adenoma of oesophagus" OR "oesophageal neoplasm" OR "oesophageal tumor" OR "oesophageal tumour" OR "oesophageal cancer" OR "oesophageal carcinoma" OR "oesophageal malignancy" OR "oesophageal adenocarcinoma" OR "oesophageal adenoma" OR "barrett neoplasm" OR "barrett tumor" OR "barrett tumour" OR "barrett cancer" OR "barrett carcinoma" OR "barrett malignancy" OR "barrett adenocarcinoma" OR "barrett adenoma" OR "barretts neoplasm" OR "barretts tumor" OR "barretts tumour" OR "barretts cancer" OR "barretts carcinoma" OR "barretts malignancy" OR "barretts adenocarcinoma" OR "barretts adenoma" OR "barrett s neoplasm" OR "barrett s tumor" OR "barrett s tumour" OR "barrett s cancer" OR "barrett s carcinoma" OR "barrett s malignancy" OR "barrett s adenocarcinoma" OR "barrett s adenoma" OR "cardia neoplasm" OR "cardia tumor" OR "cardia tumour" OR "cardia cancer" OR "cardia carcinoma" OR "cardia malignancy" OR "cardia adenocarcinoma" OR "cardia adenoma")
2 (EXPAND[Concept] "esophagus Squamous Cell carcinoma" OR EXPAND[Concept] "oesophagus Squamous Cell carcinoma" OR EXPAND[Concept] "esophageal Squamous Cell carcinoma" OR EXPAND[Concept] "oesophageal Squamous Cell carcinoma")
3 ((EXPAND[Concept] "gastroesophageal Junction" OR EXPAND[Concept] "gastrooesophageal Junction" OR EXPAND[Concept] "esophagogastric Junction" OR EXPAND[Concept] "oesophagogastric Junction") AND (Neoplasm OR tumor OR tumour OR cancer OR carcinoma OR malignancy OR adenocarcinoma OR adenoma))
4 (chemoradiotherapy OR EXPAND[Concept] "Adjuvant Radiotherapy" OR EXPAND[Concept] "chemoradio therapy" OR radiochemotherapy OR EXPAND[Concept] "radio chemotherapy" OR chemoradiation OR

EXPAND[Concept] "chemo radiation")
5 (EXPAND[Concept] ("chemotherapy" OR "chemo therapy" OR "Neoadjuvant Therapy" OR "Antineoplastic Agents" OR "Combined
Modality Therapy" OR "antineoplastic drug combination" OR "antitumor drug combination" OR "anticancer agent combination" OR
"anticancer drug combination" OR "anticancer drugs combination" OR "combined anticancer agent" OR "combined anticancer drug"))
6 (surgery OR EXPAND[Concept] "Surgical Oncology" OR Esophagectomy OR Oesophagectomy OR EXPAND[Concept] "esophago
gastrectomy" OR EXPAND[Concept] "oesophago gastrectomy" OR operative OR preoperative)
7 1 OR 2 OR 3
8 4 OR 5
9 6 AND 7 AND 8

ICTRP

esophagus neoplasm AND chemoradiotherapy AND surgery OR esophagus neoplasm AND chemotherapy AND surgery OR esophageal Squamous Cell carcinoma AND chemoradiotherapy AND surgery OR esophageal Squamous Cell carcinoma AND chemotherapy AND surgery OR gastroesophageal Junction neoplasm AND chemoradiotherapy AND surgery OR gastroesophageal Junction neoplasm AND chemotherapy AND surgery
esophagus neoplasm AND chemoradiotherapy AND surgery OR esophagus neoplasm AND chemotherapy AND surgery OR esophageal Squamous Cell carcinoma AND chemoradiotherapy AND surgery OR esophageal Squamous Cell carcinoma AND chemotherapy AND surgery OR gastroesophageal Junction neoplasm AND chemoradiotherapy AND surgery OR gastroesophageal Junction neoplasm AND chemotherapy AND surgery

eAppendix 2. Data Quality Checks

The quality of submitted Individual Participant Data (IPD) from the individual studies was assessed as follows. Inconsistencies were tried to be clarified with the respective investigators and missing data were requested. IPD were compared with the intention-to-treat population reported in publications. Datasets were checked for obvious duplicates or omissions. Plausibility of the values supplied for each variable was checked by inspecting extreme outliers. Summary statistics calculated from the dataset were compared with corresponding results in publications. OS and DFS of the different treatment groups in each trial were derived applying Kaplan-Meier and standard Cox regression analysis, and were compared with published results. Completeness and equality of follow-up in the study arms were checked by plotting a 'reverse' Kaplan-Meier curve considering censored participants as participants who incurred the outcome;[1] in addition, the median follow-up time was evaluated if it was reported in the respective publication.

eAppendix 3. Assessment of Risk of Bias in Included Studies

Two review authors (UR, JF) independently assessed the risk of bias for each included study, using the criteria outlined in the Cochrane Handbook for Systematic Reviews of Interventions[2] and version 2 of the Cochrane 'Risk of bias' tool (RoB 2) as MS Excel tool.[3] We resolved any disagreement by discussion.

We assessed the risk of bias according to the following domains:

- Bias arising from the randomization process.
- Bias due to deviations from intended interventions.
- Bias due to missing outcome data.
- Bias in measurement of the outcome.
- Bias in selection of the reported result.

The effect of interest was the effect of the assignment to the interventions at baseline, regardless of whether the interventions were actually received and adhered to as intended.

We graded each potential source of bias as "high", "some concerns" or "low", and provided a quote from the study report and justification for our judgement in the 'Risk of bias' table. We summarized the ‘Risk of bias’ judgements across studies for each of the domains listed. We considered blinding separately for different key outcomes where necessary, e.g. for unblinded outcome assessment, risk of bias for all-cause mortality may be

substantially different from risk of bias for quality of life. Where information on risk of bias related to unpublished data or correspondence with a study author, we noted this in the 'Risk of bias' table. Overall risk of bias was ascertained by using the signaling questions and algorithm provided by the RoB 2 tool.

The RoB 2 Excel tool to implement RoB 2 was used to manage the assessment of bias.

Reporting bias was assessed by comparison-adjusted funnel plots.[4]

eAppendix 4. Variables, Eligibility, and Missing Data

Variables

The following patient and trial characteristics were requested as IPD requested or retrieved from aggregate data retrieved:

- general study information: title, authors, contact address, funding source, language, publication status, year of publication, place(s) and year(s) of study conduction;
- Study design issues: in-/exclusion criteria, randomisation, risk of bias, length of study/follow-up period;
- Baseline characteristics of participants: size of intervention and comparison group, and for each group the distribution of age, sex, co-morbidity (measured, if given as World Health Organization (WHO) performance stats or American Society of Anaesthesiologists (ASA) classification), histology (AC/SCC), tumor location (esophagus, gastroesophageal junction), tumor stage (TNM and UICC stage), administration of preoperative and adjuvant therapies;
- Characteristics of the intervention: details of applied chemotherapy / chemoradiotherapy (including drug dosages, radiotherapy dosages, radiotherapy modality etc.);
- Loss to follow-up;
- Notes: funding for trial, notable conflicts of interest of trial authors.

Decision which studies were eligible for each synthesis

IPD or, if unavailable, aggregate data for each included study were verified if they contained information on each single predefined outcome. Studies were included in the respective synthesis if they contained this information.

Dealing with missing data

Analyses were performed in the intention-to-treat populations as provided in the individual studies. For missing data, we contacted investigators or study sponsors of the individual studies and asked them for the specific values. Imputation or estimation of missing data from other summary statistics was not necessary for any outcome. Our database was closed in July 2023. Any data not available at that date, either because they were not provided by the investigators as IPD or because relevant summary statistics had not been published, were not included in the analyses.

eAppendix 5. Statistical Methods

The Bayesian Network Meta-Analysis (NMA) models were fitted using Markov Chain Monte Carlo (MCMC) simulations with 100,000 iterations, 5000 burn-in iterations, and 4 independent runs (chains) using the R package gemtc.[5] Convergence was checked by using the Brooks-Gelman-Rubin method.[6] Random-effects models were used for all NMAs to account for the expected variation between trials due to clinical heterogeneity, such as inhomogeneous study populations or the variability of pre-/perioperative treatment regimens. For the between-trial heterogeneity, a half-normal prior scaled to 0.5 was used for survival outcomes as it has been recommended by Friede.[7] For binary outcomes, a half-normal prior scaled to 1 was used. A normally distributed prior with mean of 0 and standard deviation of 10,000 was used as vague prior for the relative effects.

The transitivity assumption was statistically evaluated by the heterogeneity and inconsistency. Heterogeneity was assessed by τ, that was estimated as the median standard deviation between studies observed in the posterior

distribution of the Bayesian NMA models as well as the I^2 statistic. The between study heterogeneity τ was considered with respect to log-ORs and log-HRs as reasonable (0.1-0.5), fairly high (0.5-1), and extreme (>1) according to Spiegelhalter.[8]

The node-splitting approach was used to assess inconsistency of a network of interventions based on Dias 2010[9] and van Valkenhoef 2015[10] by separating direct evidence from the network of indirect evidence. For each comparison (S vs CT, S vs. CRT, CRT vs. CT) a node-splitting model estimates the direct estimate d^{dir} taking only direct evidence into account. Information coming from the remaining network is used to obtain an indirect estimate d^{indir} for the respective treatment comparison. Consistency of each treatment comparison is indicated by the discrepancy between the direct and indirect estimate and was further assessed by comparing those (hypothesis is that $d^{dir}=d^{indir}$) as implemented in the R package gemtc (https://CRAN.R-project.org/package=gemtc). The results of the node-splitting models are presented as forest plot, that shows the direct estimate, the indirect estimate, as well as the estimate based on the network meta-analysis considering both direct and indirect evidence. In addition, the p values of the comparison between direct and indirect estimates are given, where a p value <0.05 indicates inconsistency. The node-splitting approach was applied for all endpoints where direct and indirect evidence was available for the single treatment comparisons. For none of the networks, inconsistency was detected.

Sensitivity analyses were conducted for all outcomes with respect to model assumptions and the choice of priors in order to investigate robustness of the network results.[11] A Bayesian common effect NMA model was applied as well as a normal prior with N(0, 100,000) for the relative treatment effects. The choice of the prior for the heterogeneity parameter τ for the survival outcomes was assessed using a half-normal prior with scale 1, and a vague uniform prior (0,2). For the binary outcomes, sensitivity analyses were conducted with a half-normal prior with scale 0.5, half-normal prior with scale 2, and a vague uniform prior (0,2) for τ. Sensitivity analyses based on the risk of bias assessment were not undertaken as no study was assigned to a high risk of bias.

eAppendix 6. Subgroup Analyses and Certainty Assessment

Subgroup analyses

Subgroup analyses for OS and DFS were conducted by separately (per subgroup) estimating the treatment effects in the first stage and pooling them in the second stage using the Bayesian NMA approach according to the main analyses. Therefore, the IPD population was stratified for:

- Tumor location (AEG type I vs. AEG type II/III). For stratifying patients according to tumor site we used the definition from the single trial as variable in the IPD database.
- ECOG performance status (ECOG=0 vs. ECOG=1-4).
- Age upon randomization. Initially, we planned to split the patients into three age groups (<65 years, 65 to 75 years, >75 years). Subgroups were then formed for <65 years vs. ≥65 years as there were too few patients aged >75 years.
- Sex (male vs. female).
- Surgical approach (transthoracic vs. transhiatal).
- Chemotherapeutic agents used in pre-/perioperative therapy (cisplatin/fluorouracil [5-FU] vs. other).

For all subgroup analyses except age, the HRs were adjusted for age by incorporating age as continuous covariate into the Cox regression model in the first stage. Patients with missing values for any subgroup variable were not considered for the respective subgroup analysis.

Certainty assessment

We assessed the certainty of the body of evidence from the NMA using the GRADE approach for five outcomes (overall survival [OS], disease-free survival [DFS], local and distant recurrence-free survival [RFS], postoperative morbidity, postoperative mortality, R0 resection rate).

eResults. Excluded Studies, Differences Between IPD Datasets and Published Results, and Sensitivity Analyses

Excluded studies

Important excluded studies comprise the ongoing ESOPEC[12] and RACE[13] trials which compare pre-/perioperative CT plus surgery with preoperative CRT plus surgery for AEG. The Neo-AEGIS trial, which also compares pre-/perioperative CT plus surgery with preoperative CRT plus surgery for AEG was excluded because results had only been published as conference abstract at the final search date.[14] Notable studies excluded for not comprising patients with AEG, but only with squamous cell and adenosquamous carcinoma, are the JCOG1109/NExT Study,[15] and the studies by Ancona et al.[16] and Bosset et al.[17] The trials by Macdonald et al.[18] and the CRITICS trial[19] were excluded because they randomized participants with regard to postoperative and not preoperative CRT.

Differences between IPD datasets and published results

ACCORD[20]: The quality control of IPD was already done during our previous systematic review with meta-analysis.[21] No difference between IPD and published data with respect to tumor site, resection margin, performance status, mean age, age range, and sex was found. T stage and N stage upon resection could not be directly compared between IPD and aggregate data, as the figures given in the publication were based on a different denominator.

CALGB 9781[22]: The quality control of IPD was already done during our previous systematic review with meta-analysis.[21] In the publication, results were only presented for the whole trial population (patients with both squamous cell carcinoma and adenocarcinoma); no separate results were available for patients with adenocarcinoma.

CROSS[23]: No differences between IPD and published data were found for the main outcomes.

EORTC 40954[24]: The quality control of IPD was already done during our previous systematic review with meta-analysis.[21] No differences between IPD and published data were found.

FFCD 9901[25]: There were small differences between IPD and published data for the number of patients at risk in the analysis of OS. This is probably due to longer follow up in provided IPD than in the data on which the publication was based.

MAGIC[26]: There were small differences between IPD and published data for the stages and the number of local and distant recurrences.

NeoRes[27]: There were small differences between IPD and published data for OS after 36 months and number of deaths after 60 months.

OE02[28]: There were small differences between IPD and published data in the number of deaths, median OS, patients at risk for OS, and number of recurrence sites.

POET[29]: There were small differences between IPD and published data for median age, cT stage and median OS in the CRT group.

RTOG 8911[30]: The quality control of IPD was already done during our previous systematic review with meta-analysis.[21] In the publication, results were only presented for the whole trial population (patients with both squamous cell carcinoma and adenocarcinoma); no separate published results were available for patients with adenocarcinoma.

TROG[31]: There were small differences between IPD and published data for the baseline characteristics age, ECOG, clinical stage, surgical approach, and for the number of patients: 75 in report vs. 77 in IPD, number at risk for OS, R0 resection, pCR and site of recurrence.

TROG AGITG[32]: The quality control of IPD was already done during our previous systematic review with meta-analysis.[21] In the publication, results were only presented for the whole trial population (patients with both

squamous cell carcinoma and adenocarcinoma); no separate published results were available for patients with adenocarcinoma.

Urba[33]: The quality control of IPD was already done during our previous systematic review with meta-analysis.[21] In the publication, results were only presented for the whole trial population (patients with both squamous cell carcinoma and adenocarcinoma); no separate published results were available for patients with adenocarcinoma.

Walsh[34]: The quality control of IPD was already done during our previous systematic review with meta-analysis.[21] There was a small difference in the number of patients with nodal metastasis.

In general, any differences between the IPD datasets and the published results observed were small. Sensitivity calculations showed that this had no tangible effect on the results obtained from IPD.

Sensitivity analyses

The sensitivity analyses revealed that the results of the NMA models are robust against the choice of the priors and the model assumption. The survival outcomes do not change with regard to the significance of the treatment effects (so that the 95% CrI of the HR includes the 1 or no longer includes 1). The results of the binary endpoints morbidity, pT and pN stage at resection change in some few cases where the upper or lower limit of the 95% CrI is close to 1.

eFigure 1. Network Graphs for Survival Outcomes and Binary Outcomes

Network graphs for survival outcomes (a) and binary outcomes (b). The nodes represent the interventions and the edges the treatment comparisons with the number of studies directly comparing the two interventions.

a)

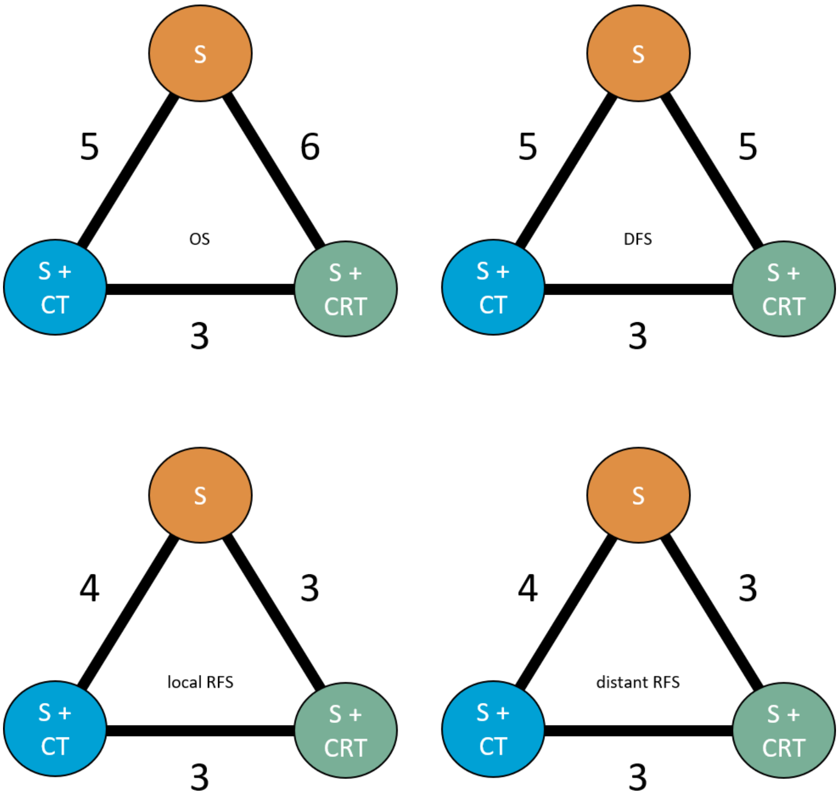

b)

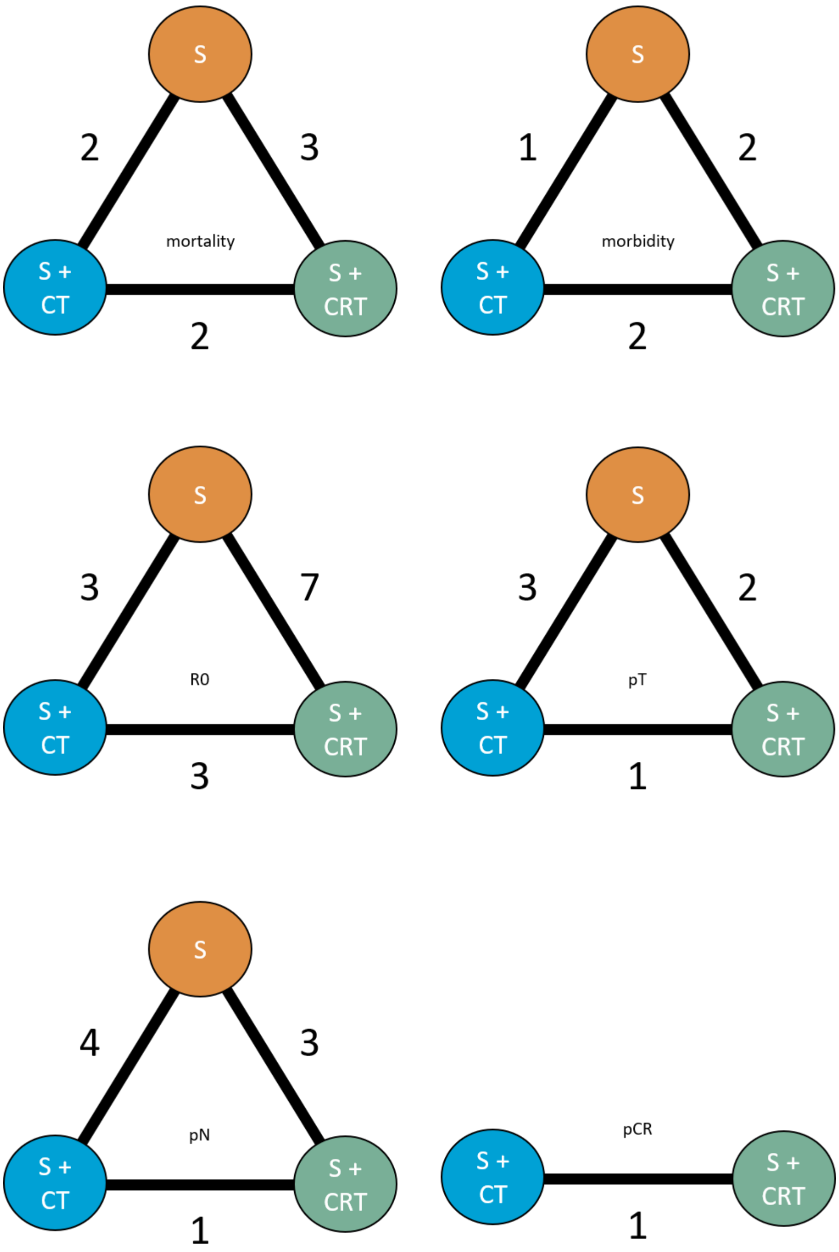
S
S + CT
S + CRT
2
3
2
mortality
S
S + CT
S + CRT
1
2
2
morbidity
S
S + CT
S + CRT
3
7
3
R0
S
S + CT
S + CRT
3
2
1
pT
S
S + CT
S + CRT
4
3
1
pN
S + CT
S + CRT
1
pCR

eFigure 2. Risk of Bias per Domain and Trials

	Randomisation process	Deviations from the intended interventions	Missing outcome data	Measurement of the outcome	Selection of the reported result	Overall
ACCORD	+	+	+	+	+	+
CALGB 9781	+	+	+	+	+	+
CROSS	+	+	+	+	+	+
EORTC 40954	+	+	+	+	+	+
FFCD 9901	+	+	+	+	+	+
MAGIC	+	+	+	+	+	+
NeoRes	+	+	+	+	?	?
OE02	+	+	+	+	+	+
POET	+	+	+	+	+	+
RTOG 8911	+	+	+	+	+	+
Tian	+	+	+	+	+	+
TOPGEAR	+	+	+	+	+	+
TROG	+	+	+	+	?	?
TROG AGITG	+	+	+	+	?	?
Urba	+	+	?	+	+	?
Walsh	+	+	+	+	+	+
Zhao	?	+	?	?	?	?

eFigure 3. Risk of Bias Summary Across Trials

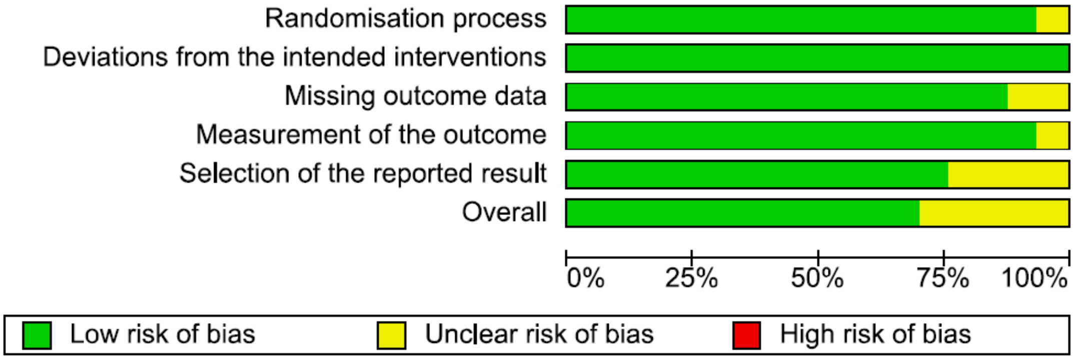

eFigure 4. Comparison-Adjusted Funnel Plots of Each Outcome

Comparison-adjusted funnel plots of each outcome with the comparison-adjusted effect size on the x-axis and the corresponding standard error on the y-axis.

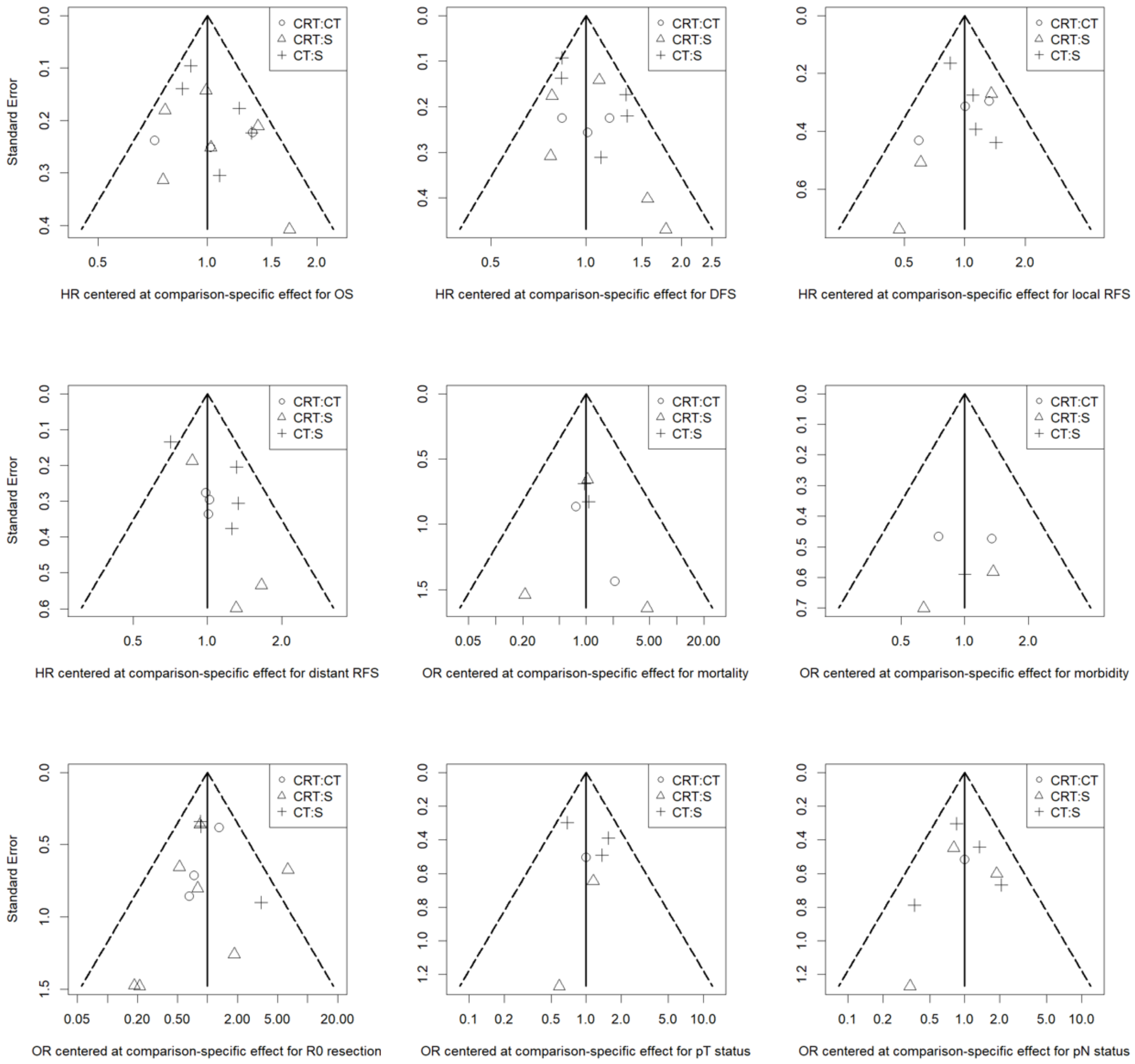

eTable. Frequency of Toxicity Events (Any Grade) in the Single Trials Reporting This Outcome

Study	preoperative treatment	number of participants	number of patients with toxicity events	relative frequency
ACCORD	CT	85	31	36.5%
RTOG 8911	CT	121	60	49.6%
FFCD 9901	CRT	30	8	26.7%
Zhao	CRT	36	6	16.7%
CROSS	CRT	130	122	93.8%

eFigure 5. Assessment of Inconsistency by the Node-Splitting Approach

The forest plots of the node-splitting analyses show the estimates of the treatment effects from the network meta-analysis model, the estimate based on the direct evidence as well as the estimate based on the indirect evidence for each treatment comparison. In addition, the numbers of included studies (St), events (Ev), and patients (Pa) of the available direct evidence are given.

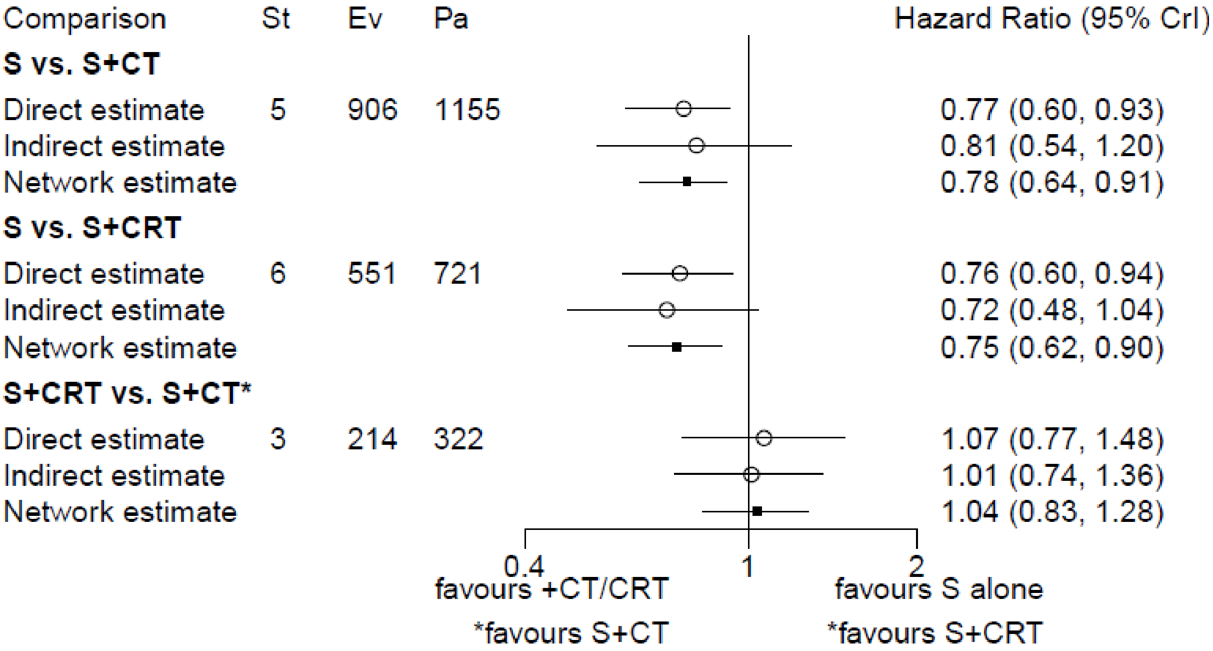

Forest plot of the node-splitting analysis of OS. The p-values of testing inconsistency for each treatment comparison by comparing direct and indirect evidence were 0.82, 0.78, 0.80. Heterogeneity of the network was estimated to be τ=0.12.

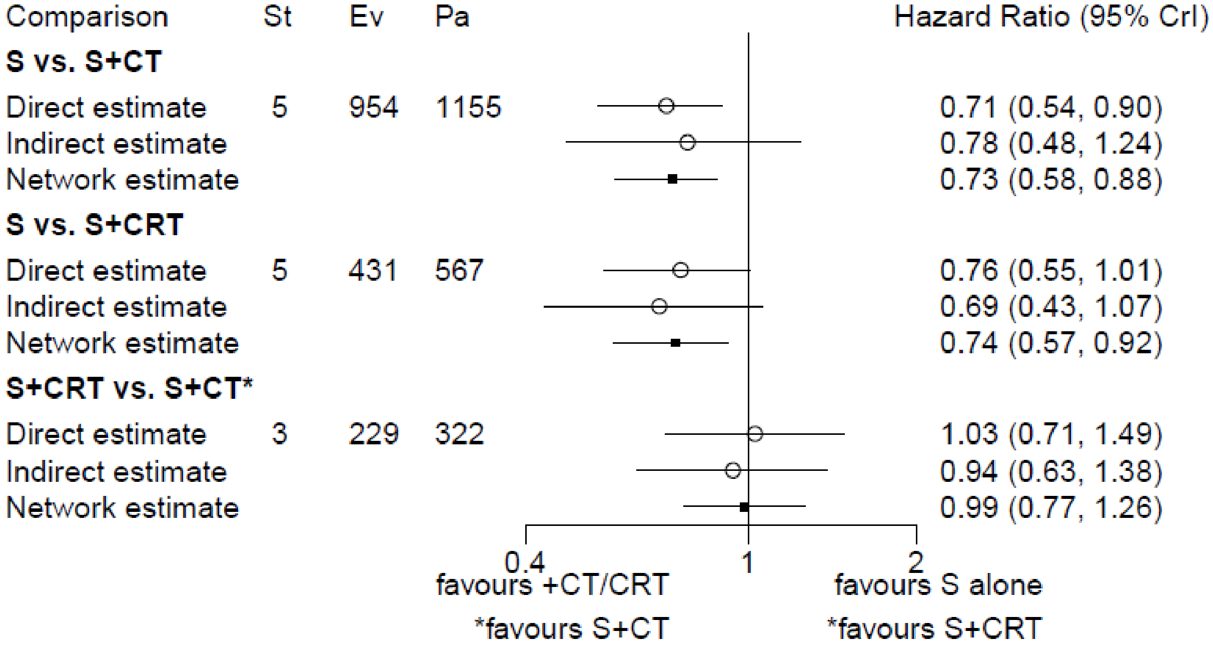

Forest plot of the node-splitting analysis of DFS. The p-values of testing inconsistency for each treatment comparison by comparing direct and indirect evidence were 0.73, 0.72, 0.73. Heterogeneity of the network was estimated to be τ=0.17.

© 2024 Ronellenfitsch U et al. *JAMA Network Open*.

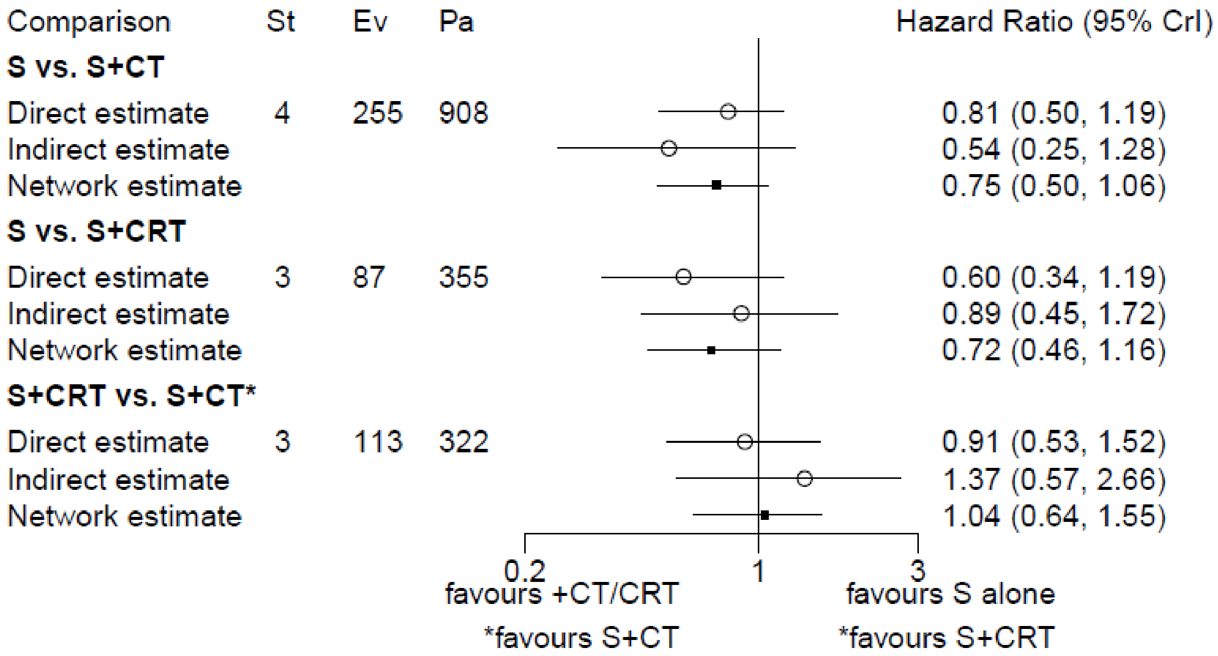

Forest plot of the node-splitting analysis of local RFS. The p-values of testing inconsistency for each treatment comparison by comparing direct and indirect evidence were 0.37, 0.37, 0.36. Heterogeneity of the network was estimated to be τ=0.26.

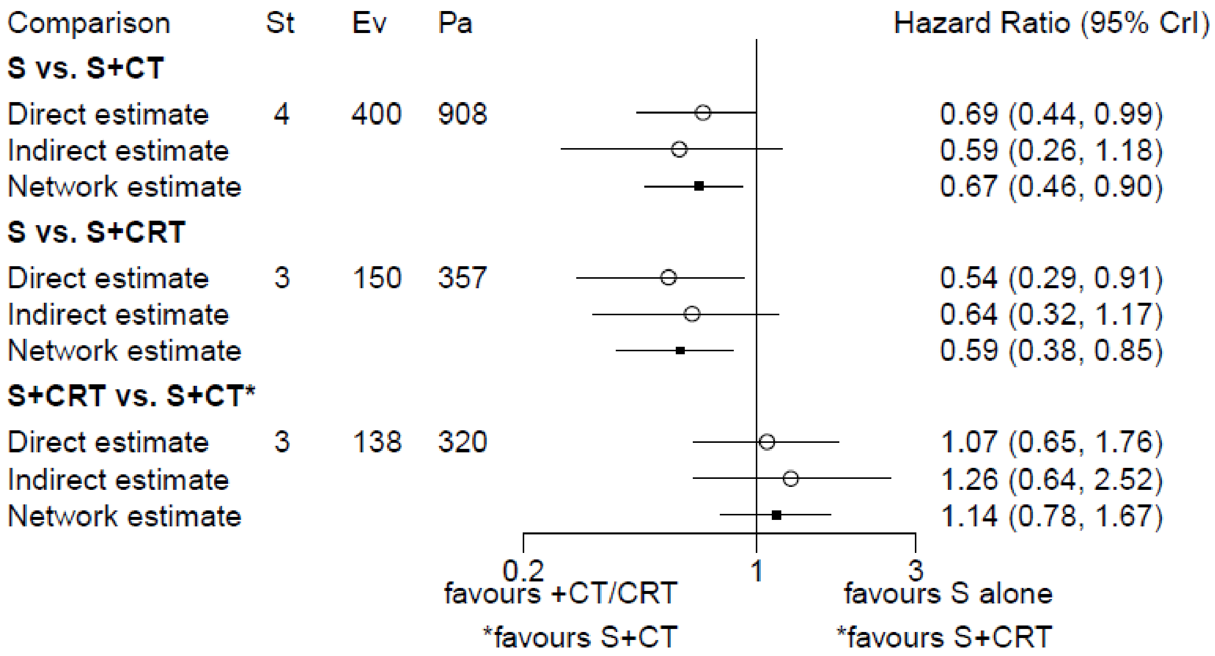

Forest plot of the node-splitting analysis of distant RFS. The p-values of testing inconsistency for each treatment comparison by comparing direct and indirect evidence were 0.67, 0.67, 0.68. Heterogeneity of the network was estimated to be τ=0.24.

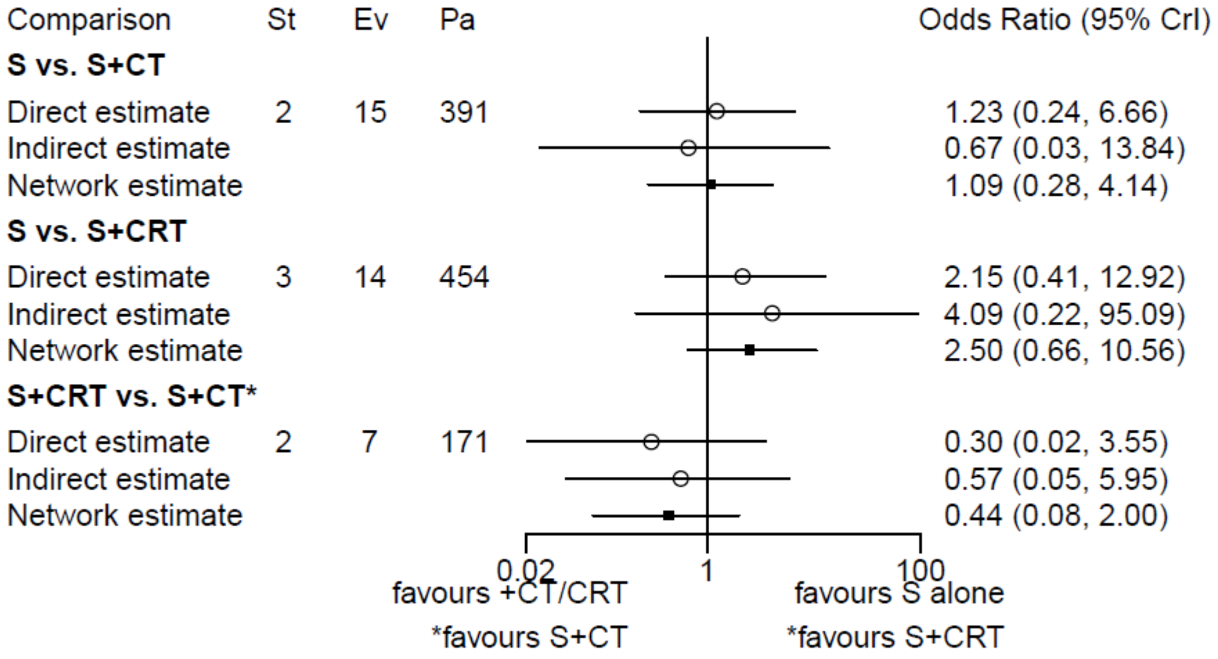

Forest plot of the node-splitting analysis of mortality. The p-values of testing inconsistency for each treatment comparison by comparing direct and indirect evidence were 0.70, 0.69, 0.71. Heterogeneity of the network was estimated to be τ=0.57.

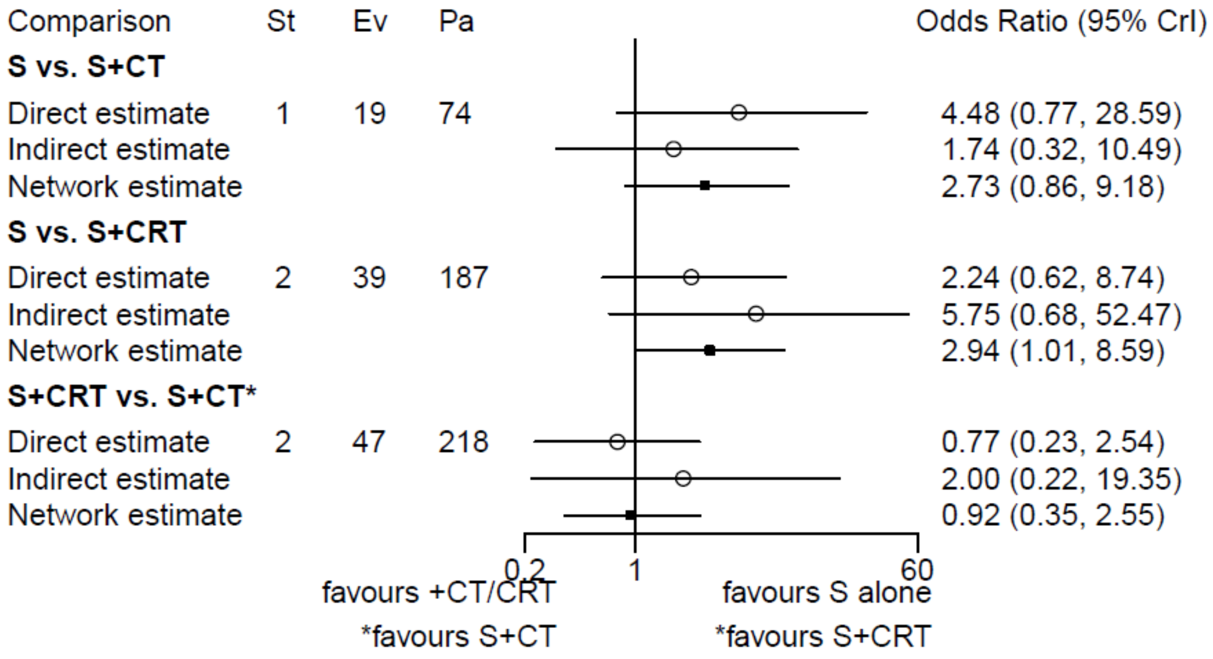

Forest plot of the node-splitting analysis of morbidity. The p-values of testing inconsistency for each treatment comparison by comparing direct and indirect evidence were 0.38, 0.38, 0.39. Heterogeneity of the network was estimated to be τ=0.48.

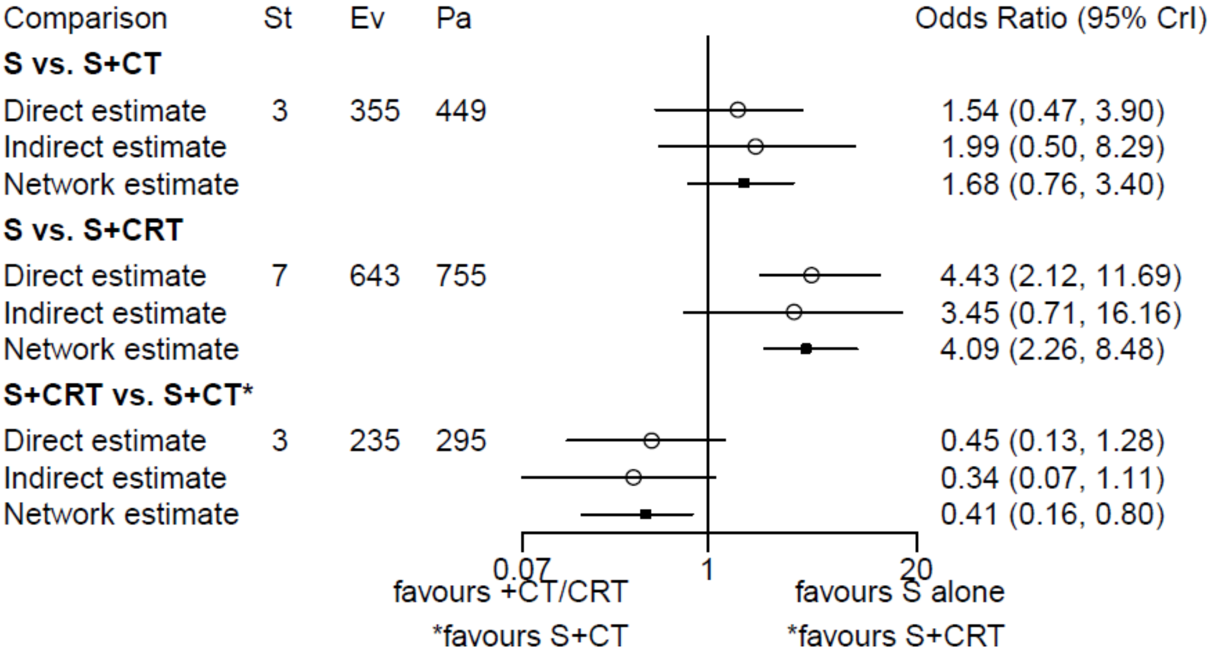

Forest plot of the node-splitting analysis of R0 resection. The p-values of testing inconsistency for each treatment comparison by comparing direct and indirect evidence were 0.73, 0.71, 0.73. Heterogeneity of the network was estimated to be τ=0.51.

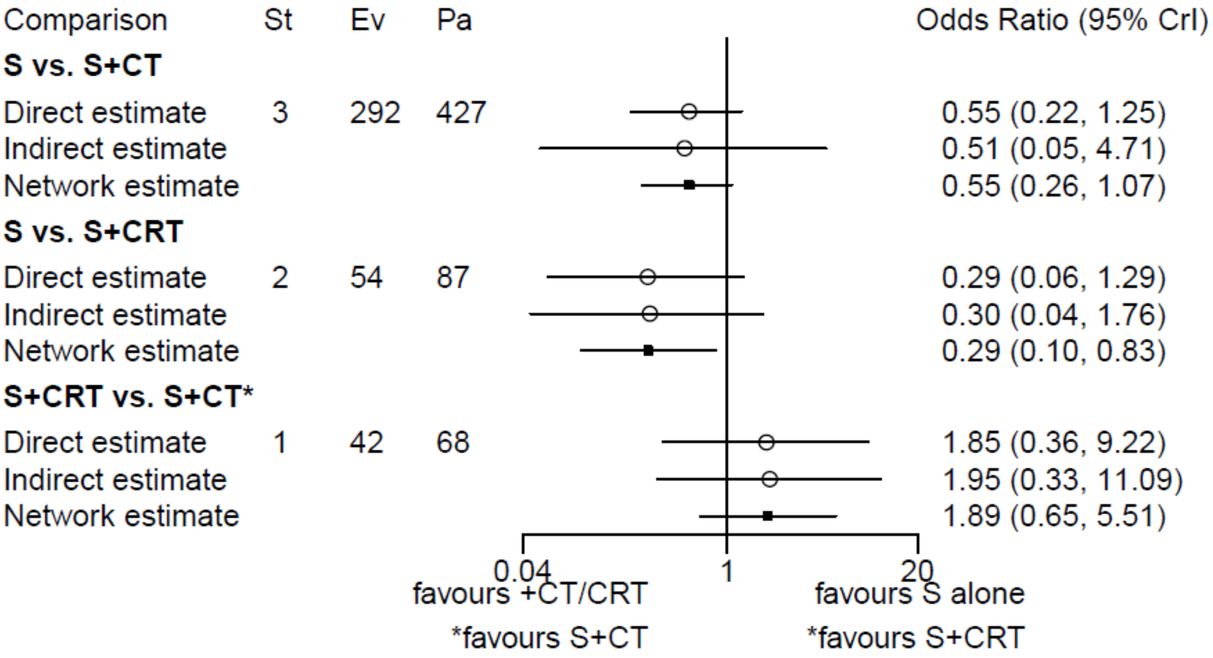

Forest plot of the node-splitting analysis of pT stage upon resection. The p-values of testing inconsistency for each treatment comparison by comparing direct and indirect evidence were 0.95, 0.96, 0.97. Heterogeneity of the network was estimated to be τ=0.41.

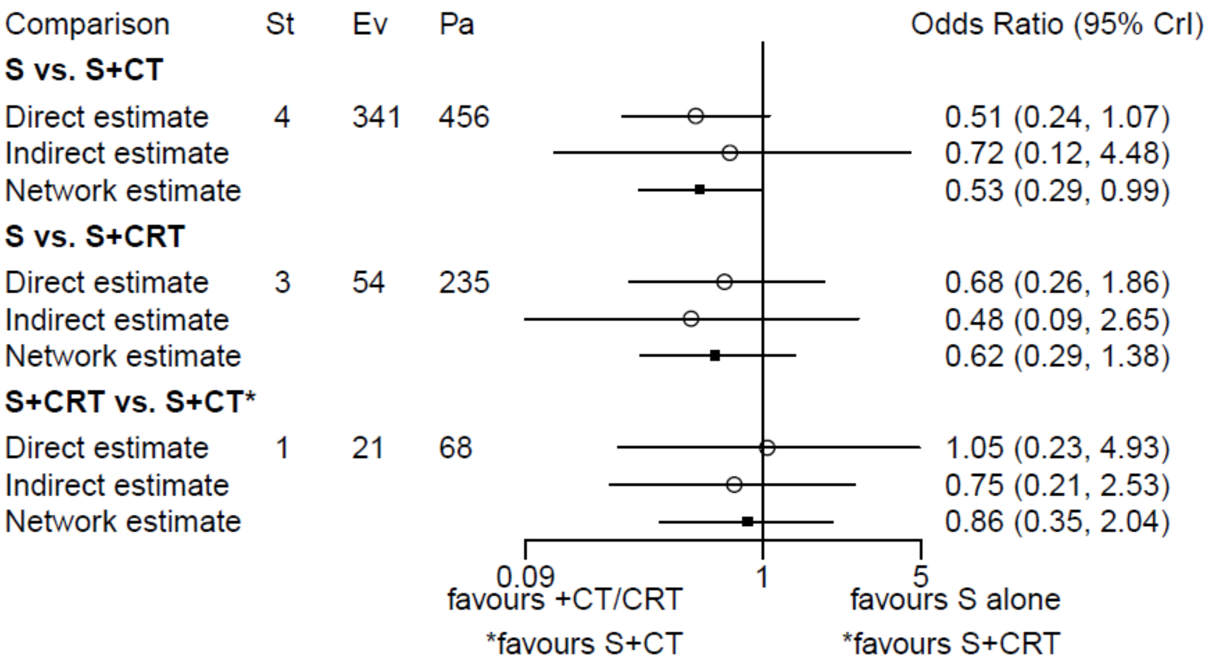

Forest plot of the node-splitting analysis of pN stage upon resection. The p-values of testing inconsistency for each treatment comparison by comparing direct and indirect evidence were 0.68, 0.70, 0.69. Heterogeneity of the network was estimated to be τ=0.35.

eFigure 6. NMA Results of the Subgroup Analyses for OS

OS - Subgroup	Studies (direct)	Patients (direct)	Events (direct)	HR (95% CrI)
**AEG type**				
**I**				
S vs. S+CT	4	673	550	0.88 (0.71 to 1.09)
S vs. S+CRT	3	422	327	0.86 (0.67 to 1.08)
S+CRT vs. S+CT*	3	218	144	1.02 (0.79 to 1.34)
**II/III**				
S vs. S+CT	5	478	352	0.68 (0.49 to 0.91)
S vs. S+CRT	2	117	87	0.50 (0.32 to 0.79)
S+CRT vs. S+CT*	3	104	70	1.34 (0.85 to 2.09)
**ECOG status**				
**0**				
S vs. S+CT	4	614	460	0.75 (0.55 to 0.96)
S vs. S+CRT	3	392	284	0.87 (0.64 to 1.18)
S+CRT vs. S+CT*	2	183	123	0.87 (0.60 to 1.19)
**1-4**				
S vs. S+CT	4	294	239	0.74 (0.45 to 1.29)
S vs. S+CRT	3	97	77	0.71 (0.35 to 1.29)
S+CRT vs. S+CT*	2	25	13	1.05 (0.51 to 2.46)
**Age**				
**< 65 years**				
S vs. S+CT	5	683	517	0.74 (0.57 to 0.94)
S vs. S+CRT	6	470	344	0.71 (0.54 to 0.90)
S+CRT vs. S+CT*	3	207	143	1.05 (0.78 to 1.41)
**>= 65 years**				
S vs. S+CT	5	472	389	0.85 (0.64 to 1.07)
S vs. S+CRT	6	251	207	0.79 (0.60 to 1.05)
S+CRT vs. S+CT*	3	115	71	1.06 (0.76 to 1.47)
**Sex**				
**Male**				
S vs. S+CT	5	1007	799	0.76 (0.62 to 0.91)
S vs. S+CRT	6	625	484	0.75 (0.61 to 0.92)
S+CRT vs. S+CT*	3	282	191	1.01 (0.79 to 1.28)
**Female**				
S vs. S+CT	5	148	107	0.84 (0.48 to 1.55)
S vs. S+CRT	4	91	65	0.72 (0.35 to 1.33)
S+CRT vs. S+CT*	3	40	23	1.16 (0.58 to 2.72)
**Surgical approach**				
**thransthoracic**				
S vs. S+CT	3	298	233	0.77 (0.54 to 1.09)
S vs. S+CRT	1	126	94	0.71 (0.45 to 1.11)
S+CRT vs. S+CT*	2	143	97	1.10 (0.71 to 1.67)
**thransabdominal**				
S vs. S+CT	3	166	115	0.48 (0.17 to 1.18)
S vs. S+CRT	1	142	97	0.73 (0.14 to 3.58)
S+CRT vs. S+CT*	0	0	0	0.66 (0.09 to 3.98)
**Chemoth. agents**				
**cisplatin/5-FU**				
S vs. S + CT/CRT	9	1470	1175	0.79 (0.64 to 0.92)
**other**				
S vs. S + CT/CRT	2	406	282	0.69 (0.42 to 1.07)

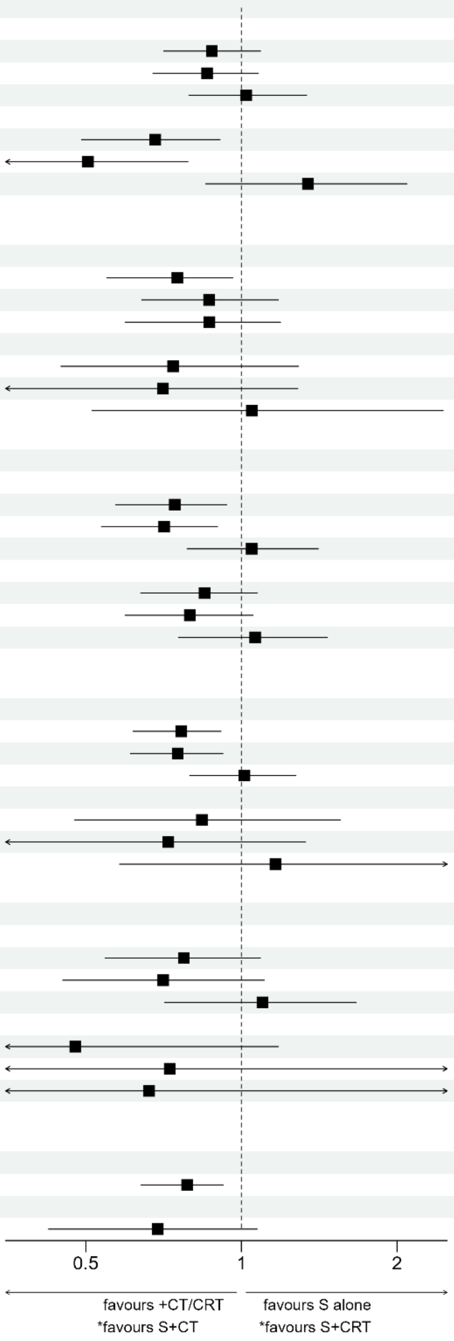

The number of studies, patients, and events are related to the direct comparison though the HR and its 95% CrI are estimated from the network using both direct and indirect evidence for each outcome.

eFigure 7. NMA Results of the Subgroup Analyses for DFS

DFS - Subgroup	Studies (direct)	Patients (direct)	Events (direct)	HR (95% CrI)
**AEG type**				
**I**				
S vs. S+CT	4	673	578	0.84 (0.67 to 1.03)
S vs. S+CRT	2	348	275	0.86 (0.66 to 1.10)
S+CRT vs. S+CT*	3	218	151	0.98 (0.75 to 1.28)
**II/III**				
S vs. S+CT	5	478	372	0.64 (0.44 to 0.90)
S vs. S+CRT	1	78	55	0.52 (0.30 to 0.91)
S+CRT vs. S+CT*	3	104	78	1.22 (0.72 to 2.02)
**ECOG status**				
**0**				
S vs. S+CT	4	614	494	0.69 (0.47 to 0.96)
S vs. S+CRT	3	392	298	0.82 (0.56 to 1.21)
S+CRT vs. S+CT*	2	183	130	0.84 (0.54 to 1.24)
**1-4**				
S vs. S+CT	4	294	245	0.66 (0.35 to 1.16)
S vs. S+CRT	3	97	82	0.68 (0.33 to 1.35)
S+CRT vs. S+CT*	2	25	16	0.98 (0.43 to 2.20)
**Age**				
**< 65 years**				
S vs. S+CT	5	683	553	0.73 (0.59 to 0.89)
S vs. S+CRT	5	389	287	0.75 (0.59 to 0.95)
S+CRT vs. S+CT*	3	207	150	0.97 (0.75 to 1.26)
**>= 65 years**				
S vs. S+CT	5	472	401	0.77 (0.55 to 1.03)
S vs. S+CRT	4	165	136	0.82 (0.56 to 1.19)
S+CRT vs. S+CT*	3	115	79	0.94 (0.63 to 1.38)
**Sex**				
**Male**				
S vs. S+CT	5	1007	840	0.73 (0.57 to 0.89)
S vs. S+CRT	5	504	388	0.77 (0.59 to 0.98)
S+CRT vs. S+CT*	3	282	204	0.95 (0.72 to 1.23)
**Female**				
S vs. S+CT	5	148	114	0.66 (0.36 to 1.26)
S vs. S+CRT	3	58	41	0.59 (0.24 to 1.27)
S+CRT vs. S+CT*	3	40	25	1.12 (0.51 to 2.82)
**Surgical approach**				
**thransthoracic**				
S vs. S+CT	3	298	243	0.71 (0.46 to 1.04)
S vs. S+CRT	1	126	97	0.64 (0.38 to 1.07)
S+CRT vs. S+CT*	2	143	104	1.10 (0.67 to 1.77)
**thransabdominal**				
S vs. S+CT	3	166	122	0.47 (0.21 to 0.95)
S vs. S+CRT	1	142	101	0.64 (0.20 to 1.82)
S+CRT vs. S+CT*	1	13	4	0.74 (0.22 to 2.45)
**Chemoth. agents**				
**cisplatin/5-FU**				
S vs. S + CT/CRT	8	1316	1091	0.76 (0.57 to 0.95)
**other**				
S vs. S + CT/CRT	2	406	294	0.63 (0.39 to 0.98)

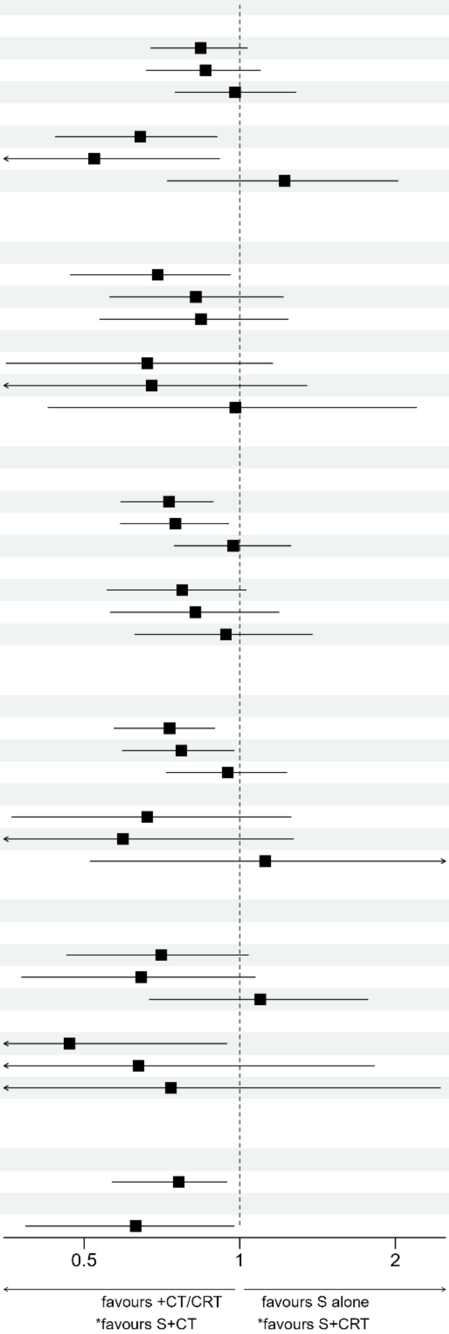

The number of studies, patients, and events are related to the direct comparison though the HR and its 95% CrI are estimated from the network using both direct and indirect evidence for each outcome.

eFigure 8. Results of the Sensitivity Analyses

a)

Outcome: OS	Studies (direct)	Patients (direct)	Events (direct)	HR (95% CrI)
**tau: half-normal(1)**				
S vs. S+CT	5	1155	906	0.78 (0.64 to 0.91)
S vs. S+CRT	6	721	551	0.75 (0.61 to 0.90)
S+CRT vs. S+CT*	3	322	214	1.04 (0.84 to 1.28)
**tau: uniform(0,2)**				
S vs. S+CT	5	1155	906	0.78 (0.64 to 0.92)
S vs. S+CRT	6	721	551	0.75 (0.62 to 0.90)
S+CRT vs. S+CT*	3	322	214	1.04 (0.84 to 1.28)
**Overall mean: N(0,10^5)**				
S vs. S+CT	5	1155	906	0.78 (0.64 to 0.92)
S vs. S+CRT	6	721	551	0.75 (0.61 to 0.89)
S+CRT vs. S+CT*	3	322	214	1.04 (0.84 to 1.29)
**Common effect model**				
S vs. S+CT	5	1155	906	0.79 (0.70 to 0.90)
S vs. S+CRT	6	721	551	0.76 (0.65 to 0.88)
S+CRT vs. S+CT*	3	322	214	1.05 (0.89 to 1.24)

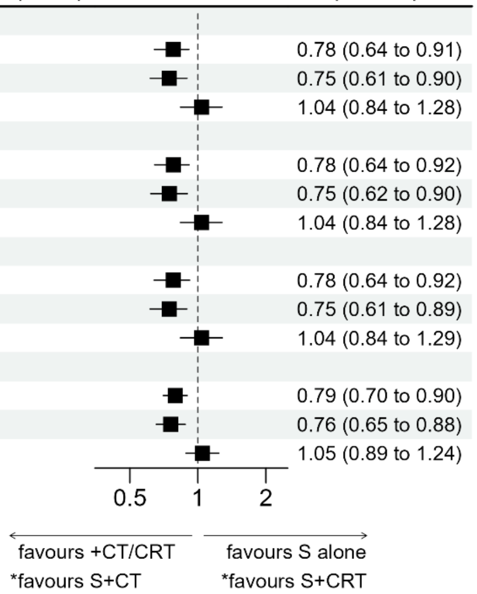

The number of studies, patients, and events are related to the direct comparison though the HR and its 95% CrI are estimated from the network using both direct and indirect evidence for each outcome.

b)

Outcome: DFS	Studies (direct)	Patients (direct)	Events (direct)	HR (95% CrI)
**tau: half-normal(1)**				
S vs. S+CT	5	1155	954	0.73 (0.58 to 0.88)
S vs. S+CRT	5	567	431	0.74 (0.58 to 0.92)
S+CRT vs. S+CT*	3	322	229	0.98 (0.76 to 1.26)
**tau: uniform(0,2)**				
S vs. S+CT	5	1155	954	0.73 (0.58 to 0.88)
S vs. S+CRT	5	567	431	0.74 (0.57 to 0.92)
S+CRT vs. S+CT*	3	322	229	0.99 (0.76 to 1.27)
**Overall mean: N(0,10^5)**				
S vs. S+CT	5	1155	954	0.73 (0.58 to 0.88)
S vs. S+CRT	5	567	431	0.74 (0.57 to 0.92)
S+CRT vs. S+CT*	3	322	229	0.99 (0.77 to 1.26)
**Common effect model**				
S vs. S+CT	5	1155	954	0.76 (0.68 to 0.86)
S vs. S+CRT	5	567	431	0.76 (0.65 to 0.89)
S+CRT vs. S+CT*	3	322	229	1.00 (0.84 to 1.19)

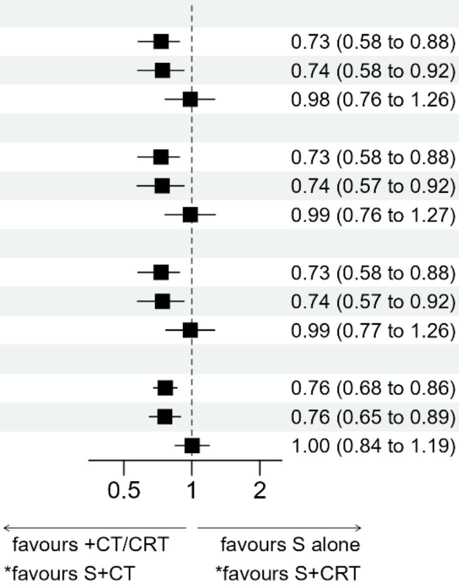

The number of studies, patients, and events are related to the direct comparison though the HR and its 95% CrI are estimated from the network using both direct and indirect evidence for each outcome.

c)

Outcome: local RFS	Studies (direct)	Patients (direct)	Events (direct)	HR (95% CrI)
**tau: half-normal(1)**				
S vs. S+CT	4	908	255	0.75 (0.50 to 1.06)
S vs. S+CRT	3	355	87	0.72 (0.47 to 1.17)
S+CRT vs. S+CT*	3	322	113	1.04 (0.64 to 1.55)
**tau: uniform(0,2)**				
S vs. S+CT	4	908	255	0.75 (0.49 to 1.08)
S vs. S+CRT	3	355	87	0.72 (0.46 to 1.17)
S+CRT vs. S+CT*	3	322	113	1.04 (0.63 to 1.55)
**Overall mean: N(0,10^5)**				
S vs. S+CT	4	908	255	0.75 (0.50 to 1.06)
S vs. S+CRT	3	355	87	0.72 (0.46 to 1.16)
S+CRT vs. S+CT*	3	322	113	1.04 (0.64 to 1.55)
**Common effect model**				
S vs. S+CT	4	908	255	0.78 (0.62 to 0.99)
S vs. S+CRT	3	355	87	0.71 (0.52 to 0.97)
S+CRT vs. S+CT*	3	322	113	1.11 (0.82 to 1.50)

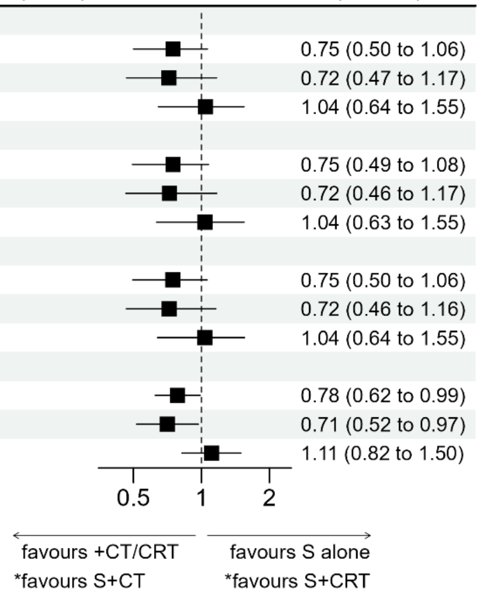

The number of studies, patients, and events are related to the direct comparison though the HR and its 95% CrI are estimated from the network using both direct and indirect evidence for each outcome.

d)

Outcome: distant RFS	Studies (direct)	Patients (direct)	Events (direct)	HR (95% CrI)
**tau: half-normal(1)**				
S vs. S+CT	4	908	400	0.67 (0.46 to 0.90)
S vs. S+CRT	3	357	150	0.59 (0.38 to 0.84)
S+CRT vs. S+CT*	3	320	138	1.14 (0.78 to 1.66)
**tau: uniform(0,2)**				
S vs. S+CT	4	908	400	0.67 (0.46 to 0.90)
S vs. S+CRT	3	357	150	0.59 (0.38 to 0.85)
S+CRT vs. S+CT*	3	320	138	1.14 (0.78 to 1.67)
**Overall mean: N(0,10^5)**				
S vs. S+CT	4	908	400	0.67 (0.46 to 0.90)
S vs. S+CRT	3	357	150	0.59 (0.38 to 0.85)
S+CRT vs. S+CT*	3	320	138	1.14 (0.78 to 1.65)
**Common effect model**				
S vs. S+CT	4	908	400	0.74 (0.62 to 0.89)
S vs. S+CRT	3	357	150	0.63 (0.49 to 0.82)
S+CRT vs. S+CT*	3	320	138	1.16 (0.90 to 1.51)

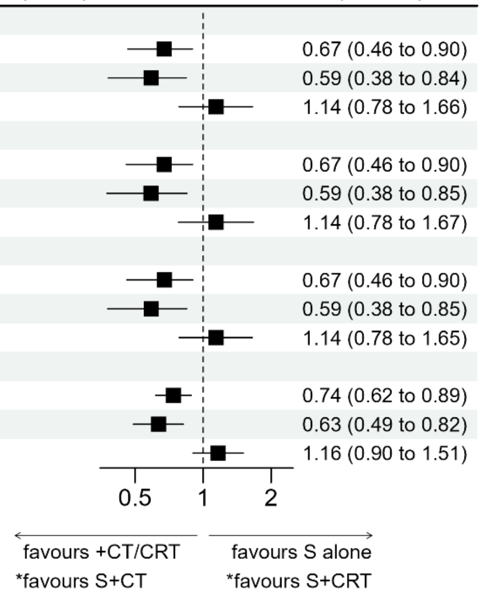

The number of studies, patients, and events are related to the direct comparison though the HR and its 95% CrI are estimated from the network using both direct and indirect evidence for each outcome.

e)

Outcome: postop. mortality	Studies (direct)	Patients (direct)	Events (direct)	OR (95% CrI)
**tau: half-normal(0.5)**				
S vs. S+CT	2	391	15	1.10 (0.37 to 3.29)
S vs. S+CRT	3	454	14	2.46 (0.81 to 7.95)
S+CRT vs. S+CT*	2	171	7	0.45 (0.12 to 1.59)
**tau: half-normal(2)**				
S vs. S+CT	2	391	15	1.09 (0.23 to 4.99)
S vs. S+CRT	3	454	14	2.52 (0.56 to 12.68)
S+CRT vs. S+CT*	2	171	7	0.43 (0.07 to 2.41)
**tau: uniform(0,2)**				
S vs. S+CT	2	391	15	1.09 (0.24 to 4.94)
S vs. S+CRT	3	454	14	2.49 (0.56 to 12.32)
NA	2	171	7	0.44 (0.07 to 2.46)
**Overall mean: N(0,10^5)**				
S vs. S+CT	2	391	15	1.09 (0.29 to 4.12)
S vs. S+CRT	3	454	14	2.48 (0.67 to 10.43)
S+CRT vs. S+CT*	2	171	7	0.44 (0.09 to 1.98)
**Common effect model**				
S vs. S+CT	2	391	15	1.09 (0.42 to 2.80)
S vs. S+CRT	3	454	14	2.41 (0.91 to 6.95)
S+CRT vs. S+CT*	2	171	7	0.45 (0.14 to 1.38)

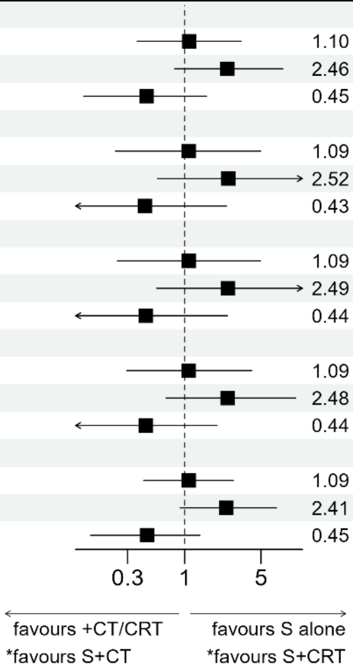

The number of studies, patients, and events are related to the direct comparison though the HR and its 95% CrI are estimated from the network using both direct and indirect evidence for each outcome.

f)

Outcome: postop. morbidity	Studies (direct)	Patients (direct)	Events (direct)	OR (95% CrI)
**tau: half-normal(0.5)**				
S vs. S+CT	1	74	19	2.69 (1.03 to 7.32)
S vs. S+CRT	2	187	39	2.92 (1.22 to 7.24)
S+CRT vs. S+CT*	2	218	47	0.92 (0.43 to 2.04)
**tau: half-normal(2)**				
S vs. S+CT	1	74	19	2.72 (0.76 to 10.27)
S vs. S+CRT	2	187	39	2.94 (0.93 to 9.65)
S+CRT vs. S+CT*	2	218	47	0.92 (0.32 to 2.81)
**tau: uniform(0,2)**				
S vs. S+CT	1	74	19	2.73 (0.72 to 11.18)
S vs. S+CRT	2	187	39	2.94 (0.88 to 10.35)
NA	2	218	47	0.93 (0.30 to 3.04)
**Overall mean: N(0,10^5)**				
S vs. S+CT	1	74	19	2.69 (0.85 to 9.03)
S vs. S+CRT	2	187	39	2.92 (1.04 to 8.36)
S+CRT vs. S+CT*	2	218	47	0.92 (0.36 to 2.49)
**Common effect model**				
S vs. S+CT	1	74	19	2.65 (1.22 to 5.91)
S vs. S+CRT	2	187	39	2.91 (1.41 to 6.18)
S+CRT vs. S+CT*	2	218	47	0.91 (0.50 to 1.65)

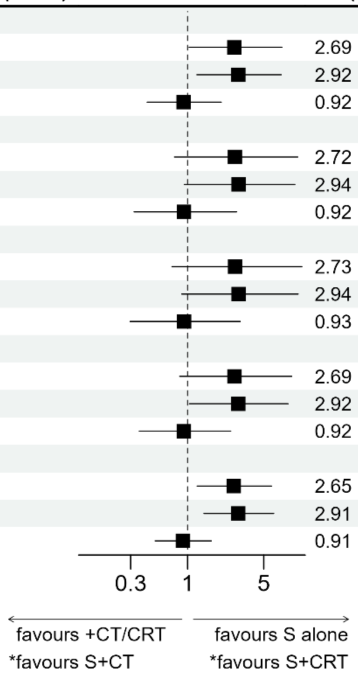

The number of studies, patients, and events are related to the direct comparison though the HR and its 95% CrI are estimated from the network using both direct and indirect evidence for each outcome.

g)

Outcome: R0 resection	Studies (direct)	Patients (direct)	Events (direct)	OR (95% CrI)
**tau: half-normal(0.5)**				
S vs. S+CT	3	449	355	1.71 (0.89 to 3.04)
S vs. S+CRT	7	755	643	4.02 (2.39 to 7.25)
S+CRT vs. S+CT*	3	295	235	0.42 (0.20 to 0.76)
**tau: half-normal(2)**				
S vs. S+CT	3	449	355	1.69 (0.73 to 3.55)
S vs. S+CRT	7	755	643	4.12 (2.21 to 8.95)
S+CRT vs. S+CT*	3	295	235	0.41 (0.16 to 0.83)
**tau: uniform(0,2)**				
S vs. S+CT	3	449	355	1.68 (0.68 to 3.73)
S vs. S+CRT	7	755	643	4.15 (2.17 to 9.46)
NA	3	295	235	0.41 (0.15 to 0.85)
**Overall mean: N(0,10^5)**				
S vs. S+CT	3	449	355	1.69 (0.76 to 3.39)
S vs. S+CRT	7	755	643	4.09 (2.28 to 8.57)
S+CRT vs. S+CT*	3	295	235	0.41 (0.17 to 0.80)
**Common effect model**				
S vs. S+CT	3	449	355	1.79 (1.20 to 2.70)
S vs. S+CRT	7	755	643	3.93 (2.64 to 5.89)
S+CRT vs. S+CT*	3	295	235	0.46 (0.29 to 0.71)

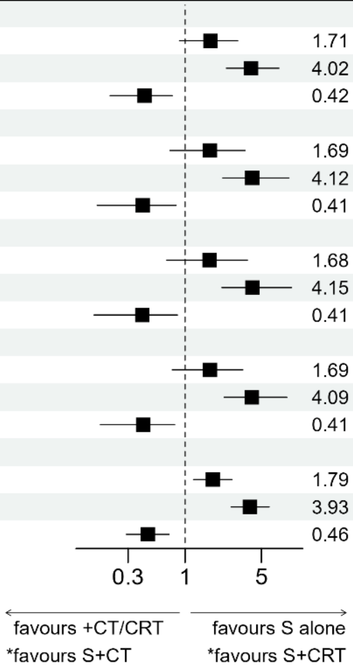

The number of studies, patients, and events are related to the direct comparison though the HR and its 95% CrI are estimated from the network using both direct and indirect evidence for each outcome.

h)

Outcome: pN stage	Studies (direct)	Patients (direct)	Events (direct)	OR (95% CrI)
**tau: half-normal(0.5)**				
S vs. S+CT	4	456	341	0.53 (0.31 to 0.91)
S vs. S+CRT	3	235	54	0.62 (0.30 to 1.26)
S+CRT vs. S+CT*	1	68	21	0.86 (0.39 to 1.89)
**tau: half-normal(2)**				
S vs. S+CT	4	456	341	0.53 (0.28 to 1.02)
S vs. S+CRT	3	235	54	0.62 (0.28 to 1.40)
S+CRT vs. S+CT*	1	68	21	0.86 (0.34 to 2.14)
**tau: uniform(0,2)**				
S vs. S+CT	4	456	341	0.53 (0.27 to 1.08)
S vs. S+CRT	3	235	54	0.62 (0.27 to 1.46)
NA	1	68	21	0.86 (0.33 to 2.21)
**Overall mean: N(0,10^5)**				
S vs. S+CT	4	456	341	0.53 (0.29 to 0.98)
S vs. S+CRT	3	235	54	0.62 (0.28 to 1.33)
S+CRT vs. S+CT*	1	68	21	0.87 (0.36 to 2.07)
**Common effect model**				
S vs. S+CT	4	456	341	0.54 (0.35 to 0.81)
S vs. S+CRT	3	235	54	0.62 (0.34 to 1.11)
S+CRT vs. S+CT*	1	68	21	0.86 (0.45 to 1.64)

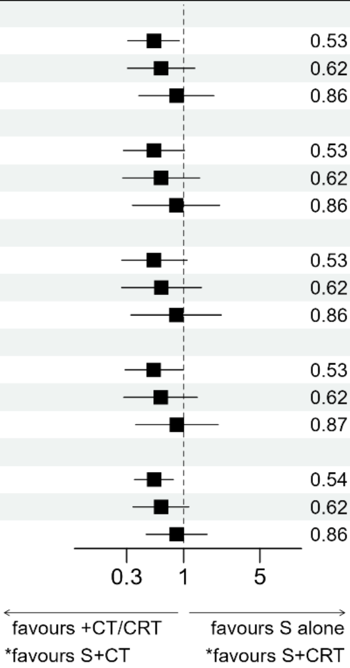

The number of studies, patients, and events are related to the direct comparison though the HR and its 95% CrI are estimated from the network using both direct and indirect evidence for each outcome.

i)

Outcome: pT stage	Studies (direct)	Patients (direct)	Events (direct)	OR (95% CrI)
**tau: half-normal(0.5)**				
S vs. S+CT	3	427	292	0.55 (0.30 to 0.97)
S vs. S+CRT	2	87	54	0.29 (0.11 to 0.74)
S+CRT vs. S+CT*	1	68	42	1.90 (0.73 to 4.95)
**tau: half-normal(2)**				
S vs. S+CT	3	427	292	0.55 (0.25 to 1.11)
S vs. S+CRT	2	87	54	0.29 (0.09 to 0.86)
S+CRT vs. S+CT*	1	68	42	1.88 (0.60 to 5.77)
**tau: uniform(0,2)**				
S vs. S+CT	3	427	292	0.54 (0.23 to 1.16)
S vs. S+CRT	2	87	54	0.29 (0.09 to 0.91)
NA	1	68	42	1.88 (0.55 to 6.00)
**Overall mean: N(0,10^5)**				
S vs. S+CT	3	427	292	0.55 (0.26 to 1.07)
S vs. S+CRT	2	87	54	0.29 (0.10 to 0.83)
S+CRT vs. S+CT*	1	68	42	1.89 (0.63 to 5.51)
**Common effect model**				
S vs. S+CT	3	427	292	0.57 (0.38 to 0.85)
S vs. S+CRT	2	87	54	0.30 (0.13 to 0.65)
S+CRT vs. S+CT*	1	68	42	1.91 (0.89 to 4.22)

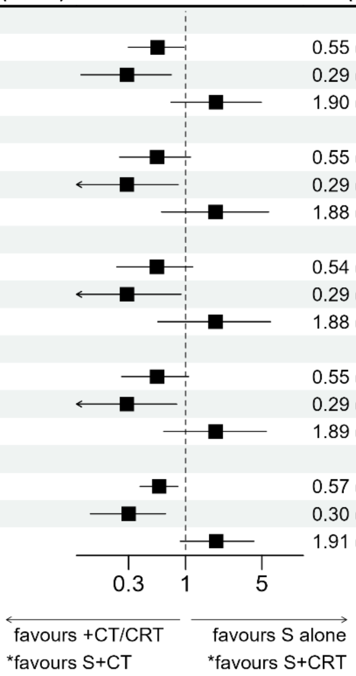

The number of studies, patients, and events are related to the direct comparison though the HR and its 95% CrI are estimated from the network using both direct and indirect evidence for each outcome.

eReferences.

1. Stewart LA, Cochrane Working Group On Meta-Analysis Using Individual Patient D. Practical methodology of meta-analyses (overviews) using updated individual patient data. *Statistics in Medicine*. 1995;14(19):2057-2079.
2. Higgins JPT TJ, Chandler J, Cumpston M, Li T, Page MJ, Welch VA. *Cochrane Handbook for Systematic Reviews of Interventions version 6.4 (updated August 2023)*. Cochrane; 2023. www.training.cochrane.org/handbook
3. Sterne JAC, Savović J, Page MJ, et al. RoB 2: a revised tool for assessing risk of bias in randomised trials. *Bmj*. 2019;366:l4898.
4. Chaimani A, Salanti G. Using network meta-analysis to evaluate the existence of small-study effects in a network of interventions. *Research Synthesis Methods*. 2012;3(2):161-176.
5. van Valkenhoef G KJ. Bayesian Methods. R package version 1.0-2. https://CRAN.R-project.org/package=gemtc
6. Stephen P, Brooks, Andrew, Gelman. General methods for monitoring convergence of iterative simulations. *Journal of Computational and Graphical Statistics*. 1998;7:434-455.
7. Friede T, Röver C, Wandel S, Neuenschwander B. Meta-analysis of few small studies in orphan diseases. *Res Synth Methods*. 2017;8(1):79-91.
8. Spiegelhalter DJ, Abrams KR, Myles JP. *Bayesian approaches to clinical trials and health-care evaluation*. vol 13. John Wiley & Sons; 2004.
9. Dias S, Welton NJ, Caldwell DM, Ades AE. Checking consistency in mixed treatment comparison meta-analysis. *Statistics in Medicine*. 2010;29(7-8):932-944.
10. van Valkenhoef G, Dias S, Ades AE, Welton NJ. Automated generation of node-splitting models for assessment of inconsistency in network meta-analysis. *Research Synthesis Methods*. 2016;7(1):80-93.
11. Bafeta A, Trinquart L, Seror R, Ravaud P. Reporting of results from network meta-analyses: methodological systematic review. *Bmj*. 2014;348:g1741.
12. Hoeppner J, Lordick F, Brunner T, et al. ESOPEC: prospective randomized controlled multicenter phase III trial comparing perioperative chemotherapy (FLOT protocol) to neoadjuvant chemoradiation (CROSS protocol) in patients with adenocarcinoma of the esophagus (NCT02509286). *BMC Cancer*. 2016;16(1):503.
13. Lorenzen S, Biederstädt A, Ronellenfitsch U, et al. RACE-trial: neoadjuvant radiochemotherapy versus chemotherapy for patients with locally advanced, potentially resectable adenocarcinoma of the gastroesophageal junction - a randomized phase III joint study of the AIO, ARO and DGAV. *BMC Cancer*. 2020;20(1):886.
14. Reynolds JV, Preston SR, O'Neill B, et al. Trimodality therapy versus perioperative chemotherapy in the management of locally advanced adenocarcinoma of the oesophagus and oesophagogastric junction (Neo-AEGIS): an open-label, randomised, phase 3 trial. *Lancet Gastroenterol Hepatol*. 2023;8(11):1015-1027.
15. Kato K, Ito Y, Daiko H, et al. A randomized controlled phase III trial comparing two chemotherapy regimen and chemoradiotherapy regimen as neoadjuvant treatment for locally advanced esophageal cancer, JCOG1109 NExT study. *Journal of Clinical Oncology*. 2022;40(4_suppl):238-238.
16. Ancona E, Ruol A, Castoro C, et al. First-Line Chemotherapy Improves the Resection Rate and Long-Term Survival of Locally Advanced (T4, any N, M0) Squamous Cell Carcinoma of the Thoracic Esophagus: Final Report on 163 Consecutive Patients With 5-Year Follow-Up. *Annals of Surgery*. 1997;226(6):714-724.
17. Bosset J-F, Gignoux M, Triboulet J-P, et al. Chemoradiotherapy Followed by Surgery Compared with Surgery Alone in Squamous-Cell Cancer of the Esophagus. *New England Journal of Medicine*. 1997;337(3):161-167.
18. Macdonald JS, Smalley SR, Benedetti J, et al. Chemoradiotherapy after surgery compared with surgery alone for adenocarcinoma of the stomach or gastroesophageal junction. *N Engl J Med*. 2001;345(10):725-30.
19. Cats A, Jansen EPM, van Grieken NCT, et al. Chemotherapy versus chemoradiotherapy after surgery and preoperative chemotherapy for resectable gastric cancer (CRITICS): an international, open-label,

randomised phase 3 trial. *The Lancet Oncology*. 2018;19(5):616-628.

20. Ychou M, Boige V, Pignon JP, et al. Perioperative chemotherapy compared with surgery alone for resectable gastroesophageal adenocarcinoma: an FNCLCC and FFCD multicenter phase III trial. *J Clin Oncol*. 2011;29(13):1715-21.
21. Ronellenfitsch U, Schwarzbach M, Hofheinz R, et al. Perioperative chemo(radio)therapy versus primary surgery for resectable adenocarcinoma of the stomach, gastroesophageal junction, and lower esophagus. *Cochrane Database Syst Rev*. 2013;(5):Cd008107.
22. Tepper J, Krasna MJ, Niedzwiecki D, et al. Phase III trial of trimodality therapy with cisplatin, fluorouracil, radiotherapy, and surgery compared with surgery alone for esophageal cancer: CALGB 9781. *J Clin Oncol*. 2008;26(7):1086-92.
23. Eyck BM, van Lanschot JJB, Hulshof M, et al. Ten-Year Outcome of Neoadjuvant Chemoradiotherapy Plus Surgery for Esophageal Cancer: The Randomized Controlled CROSS Trial. *J Clin Oncol*. 2021;39(18):1995-2004.
24. Schuhmacher C, Gretschel S, Lordick F, et al. Neoadjuvant chemotherapy compared with surgery alone for locally advanced cancer of the stomach and cardia: European Organisation for Research and Treatment of Cancer randomized trial 40954. *J Clin Oncol*. 2010;28(35):5210-8.
25. Mariette C, Dahan L, Mornex F, et al. Surgery alone versus chemoradiotherapy followed by surgery for stage I and II esophageal cancer: final analysis of randomized controlled phase III trial FFCD 9901. *J Clin Oncol*. 2014;32(23):2416-22.
26. Cunningham D, Allum WH, Stenning SP, et al. Perioperative chemotherapy versus surgery alone for resectable gastroesophageal cancer. *N Engl J Med*. 2006;355(1):11-20.
27. von Döbeln GA, Klevebro F, Jacobsen AB, et al. Neoadjuvant chemotherapy versus neoadjuvant chemoradiotherapy for cancer of the esophagus or gastroesophageal junction: long-term results of a randomized clinical trial. *Dis Esophagus*. 2019;32(2)
28. Allum WH, Stenning SP, Bancewicz J, Clark PI, Langley RE. Long-term results of a randomized trial of surgery with or without preoperative chemotherapy in esophageal cancer. *J Clin Oncol*. 2009;27(30):5062-7.
29. Stahl M, Walz MK, Riera-Knorrenschild J, et al. Preoperative chemotherapy versus chemoradiotherapy in locally advanced adenocarcinomas of the oesophagogastric junction (POET): Long-term results of a controlled randomised trial. *Eur J Cancer*. 2017;81:183-190.
30. Kelsen DP, Winter KA, Gunderson LL, et al. Long-term results of RTOG trial 8911 (USA Intergroup 113): a random assignment trial comparison of chemotherapy followed by surgery compared with surgery alone for esophageal cancer. *J Clin Oncol*. 2007;25(24):3719-25.
31. Burmeister BH, Thomas JM, Burmeister EA, et al. Is concurrent radiation therapy required in patients receiving preoperative chemotherapy for adenocarcinoma of the oesophagus? A randomised phase II trial. *Eur J Cancer*. 2011;47(3):354-60.
32. Burmeister BH, Smithers BM, Gebski V, et al. Surgery alone versus chemoradiotherapy followed by surgery for resectable cancer of the oesophagus: a randomised controlled phase III trial. *Lancet Oncol*. 2005;6(9):659-68.
33. Urba SG, Orringer MB, Turrisi A, Iannettoni M, Forastiere A, Strawderman M. Randomized trial of preoperative chemoradiation versus surgery alone in patients with locoregional esophageal carcinoma. *J Clin Oncol*. 2001;19(2):305-13.
34. Walsh TN, Noonan N, Hollywood D, Kelly A, Keeling N, Hennessy TP. A comparison of multimodal therapy and surgery for esophageal adenocarcinoma. *N Engl J Med*. 1996;335(7):462-7.

© 2024 Ronellenfitsch U et al. *JAMA Network Open*.
