# Supplementary material for: Preoperative Chemoradiotherapy vs Chemotherapy for Adenocarcinoma of the Esophagogastric Junction: A Network Meta-Analysis
Source: JAMA Netw Open. 2024 Aug 2;7(8):e2425581. doi: 10.1001/jamanetworkopen.2024.25581 (PMC11297377; doi:10.1001/jamanetworkopen.2024.25581)
Supplement: Supplement 2. — Data Sharing Statement [file jamanetwopen-e2425581-s002.pdf]

Data Sharing Statement

Ronellenfitsch. Preoperative Chemoradiotherapy vs Chemotherapy for Adenocarcinoma of the Esophagogastric Junction. *JAMA Netw Open*. Published August 02, 2024.
doi:10.1001/jamanetworkopen.2024.25581

Data
Data available: No

Additional Information
Explanation for why data not available: The data is the property of the original trialists, with whom data sharing agreements would need to be reached.
